# Active site structure and absorption spectrum of channelrhodopsin-2 wild-type and C128T mutant†
†Electronic supplementary information (ESI) available: Details about molecular dynamics simulations and excited-state calculations; comparison between SORCI and OM2/MRCI excited-state calculations on QM/MM optimized ChR2-WT structures; characterization of the structural transition for ChR2-WT and ChR2-C128T; Cartesian coordinates of representative BR and ChR2 active site structures; details about the metadynamics runs; Gaussian fit parameters. Movie 1: Switch between different RSBH^+^ hydrogen-bonding patterns and E123 configurations in ChR2-C128T. Movie 2: Switch between different E123 configurations in ChR2-WT. Movie 3: Switch between different RSBH^+^ hydrogen-bonding patterns in ChR2-WT. See DOI: 10.1039/c6sc00468g


**DOI:** 10.1039/c6sc00468g

**Published:** 2016-02-26

**Authors:** Yanan Guo, Franziska E. Beyle, Beatrix M. Bold, Hiroshi C. Watanabe, Axel Koslowski, Walter Thiel, Peter Hegemann, Marco Marazzi, Marcus Elstner

**Affiliations:** a Department of Theoretical Chemical Biology , Institute of Physical Chemistry , KIT , Kaiserstrasse 12 , 76131 Karlsruhe , Germany . Email: marcus.elstner@kit.edu ; Email: marco.marazzi@univ-lorraine.fr; b Research Center for Advanced Science and Technology , The University of Tokyo , 4-6-1 Komaba, Meguro-ku , Tokyo 153-8904 , Japan; c Max-Planck-Institut für Kohlenforschung , Kaiser-Wilhelm-Platz 1 , 45470 Mülheim an der Ruhr , Germany; d Institute of Biology , Experimental Biophysics , Humboldt-Universität , Invalidenstraße 42 , D-10115 Berlin , Germany

## Abstract

In spite of considerable interest, the active site of channelrhodopsin still lacks a detailed atomistic description, the understanding of which could strongly enhance the development of novel optogenetics tools. We present a computational study combining different state-of-the-art techniques, including hybrid quantum mechanics/molecular mechanics schemes and high-level quantum chemical methods, to properly describe the hydrogen-bonding pattern between the retinal chromophore and its counterions in channelrhodopsin-2 Wild-Type and C128T mutant. Especially, we show by extensive ground state dynamics that the active site, containing a glutamic acid (E123) and a water molecule, is highly dynamic, sampling three different hydrogen-bonding patterns. This results in a broad absorption spectrum that is representative of the different structural motifs found. A comparison with bacteriorhodopsin, characterized by a pentagonal hydrogen-bonded active site structure, elucidates their different absorption properties.

## Abstract 2

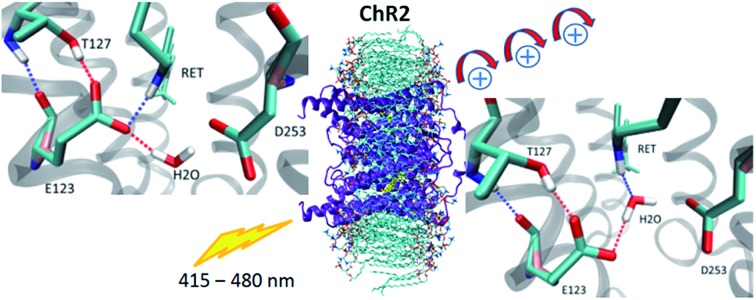
We show by extensive ground state and absorption spectra simulations that the channelrhodopsin-2 active site samples three different hydrogen-bonding patterns.

## Introduction

**Fig. 1:**
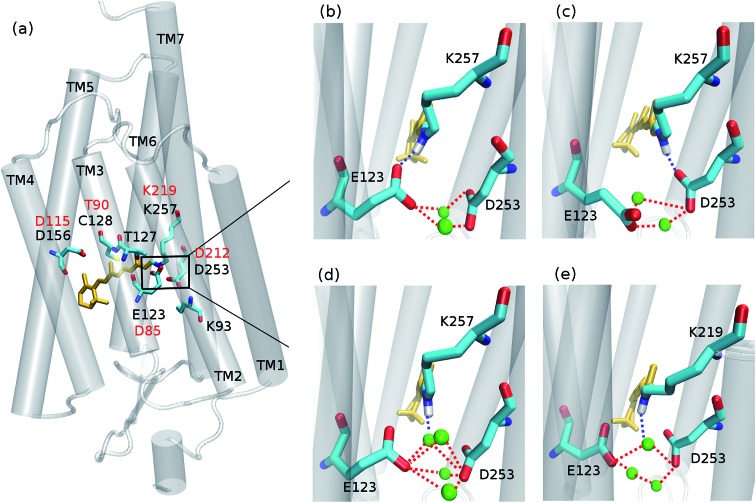
(a) The seven-helices (TM1–TM7) structure of ChR2 homology model using C1C2 crystal structure as the template. The retinal is displayed in orange. The corresponding residues in BR are labeled in red. Zoom in the active site (the rectangle area) showing the hydrogen-bonding patterns sampled in the present study: (b) –RSBH^+^···^–^O–(E123), (c) –RSBH^+^···^–^O–(D253), and (d) –RSBH^+^···OH_2_. (e) Active site of BR: a pentagonal cluster is formed between the RSBH^+^, D85, D212, and three water molecules. The dashed lines depict hydrogen bonds, and the water molecules are shown as green balls.

Since its introduction^1^–^3^ optogenetics offers to scientists the possibility to control neural tissues at the molecular level with unprecedented high temporal and spatial precision. Among the different microbial rhodopsins used for optogenetic applications, most of the attention was devoted to channelrhodopsin (ChR) (Fig. 1a). ChR irradiation triggers isomerization of the retinal chromophore and a series of biochemical steps that finally lead to the opening of a channel for passive transport of protons and cations across a membrane.^4^ ChR1 and ChR2 are the firstly discovered ChRs, which naturally occur in *Chlamydomonas reinhardtii*.^5^,^6^ Most biophysical and optogenetical researches address ChR2 because of its easier expression in host organisms.^7^ Moreover, the absorption spectrum of ChR1 is pH-dependent, while being pH-independent for ChR2 in the range pH 4–9.^8^

The understanding of the absorption properties based on an appropriate molecular description of the active site is fundamental to propose and achieve bathochromic (*i.e.* red) shifted ChRs – preferred for biological applications – and is also crucial as a starting point for an accurate investigation of the photocycle.^9^–^11^ Different studies have attempted to clarify the hydrogen-bonding network between the protonated Schiff base (RSBH^+^) and the surrounding media, but a consensus view is still lacking.^12^

Moreover, the retinal configuration in the dark adapted state is still a matter of debate. Resonance Raman and FT-IR difference spectroscopy at room temperature show bands for both, all-*trans*,15-*anti* and 13-*cis*,15-*syn* retinals (from now on called all-*trans* and 13-*cis*, respectively).^13^,^14^ Bruun *et al.*^15^ report the pure all-*trans* retinal in the initial dark adapted (IDA) state of ChR2, however, a minor 13-*cis* retinal contribution was found only for the so-called apparent dark adapted state, mainly due to the photoisomerization of all-*trans* retinal under illumination. Further, Becker-Baldus *et al.*^16^ report as well a pure all-*trans* retinal through a solid-state NMR study at 100 K. These studies show that only a pure all-*trans* retinal conformation appears after several hours in the darkness. On the other hand, the dark adapted state for smaller recovery times seems to be a mixture of all-*trans* and 13-*cis* retinal conformers.

At present, a single X-ray structure is available only for a C1C2 chimera, a hybrid composed of helices 1–5 from ChR1 and helices 6–7 from ChR2, with chromophoric properties similar to those of ChR1.^17^ In this structure the side-chain oxygen atoms of the counterions E123 and D253 are close to the retinal Schiff base nitrogen (3.4 and 3.0 Å, respectively). Empirical p*K*_a_ calculations^17^ and hybrid quantum mechanics/molecular mechanics (QM/MM) simulations^18^,^19^ suggested that in the C1C2 chimera, as well as in ChR1, the D253 residue is negatively charged and the E123 residue is neutral (at least in the crystal condition), while in ChR2 wild-type (ChR2-WT) both D253 and E123 are negatively charged, as also evidenced by spectroscopic measurements.^13^,^20^–^22^ Besides, in C1C2 the negatively charged E123 side chain prefers to form a hydrogen bond with the T127 hydroxyl group.^18^ Furthermore, several C1C2 site-specific mutants were identified by electrostatic analysis that can produce shifts of the absorption maximum while avoiding large side-effects on the channel activity.^23^

Concerning ChR2, early studies proposed E123 as the primary acceptor of the retinal proton (Fig. 1b).^24^,^25^ However, recently Heberle *et al.* proposed on the basis of time-resolved Fourier transform infrared (FTIR) spectroscopy that the proton acceptor in ChR2 is also D253, therefore identifying this residue as prominent RSBH^+^ counterion (Fig. 1c),^26^ whereas Kuhne *et al.*^22^ showed by time-resolved FTIR that in ChR2 both residues E123 and D253 are protonated, either simultaneously or alternately, during formation of the P390 photocycle intermediate.

Water might also play an important role in the ChR active site. In the C1C2 chimera only one water molecule (w619) was resolved near the RSBH^+^, at a distance (4.4 Å) too large to be considered as direct proton acceptor from the retinal.^17^ Nevertheless in ChR2, QM/MM simulations^19^ point to a possible direct hydrogen bond between the RSBH^+^ and a water molecule (Fig. 1d), consistent with the down-shifted resonance Raman spectra of the retinal C=N–H vibration.^13^,^15^

Here, we study the dark-state active site structure of ChR2-WT and C128T mutant (ChR2-C128T) using extended classical molecular dynamics (MD) and QM/MM simulations. The point mutation slows down the photocycle, since the conducting (*i.e.* opened) state, P520, is accumulated for several seconds upon illumination,^25^,^27^–^30^ while the wild-type accumulates the intermediate P480 on a second time range, corresponding to a non-conducting (*i.e.* closed) desensitized state.^24^,^31^,^32^

We first performed extensive QM/MM simulations to examine the hydrogen bonds of the RSBH^+^ with nearby residues, mainly E123 and D253 (see Fig. 1 and 3), and thus to find out which hydrogen-bonding patterns are expected to play a major role in the overall active site description.

Subsequently excited-state calculations were carried out in order to simulate absorption spectra, by applying two different techniques: (i) *ab initio* high-level quantum mechanics on a set of QM/MM optimized structures and (ii) semi-empirical multi-reference configuration interaction on a large ensemble of QM/MM trajectory snapshots. This allowed us to model the broad wavelength range typical of ChR2 proteins, as well as the contribution of the identified structural motifs to the overall spectrum.

The same ground- and excited-state strategies were applied to bacteriorhodopsin (BR), for which the active site structure is known,^33^–^38^ in order to compare its spectral characteristics with those of ChR2.

## Methods

### Rhodopsin models

**Fig. 2:**
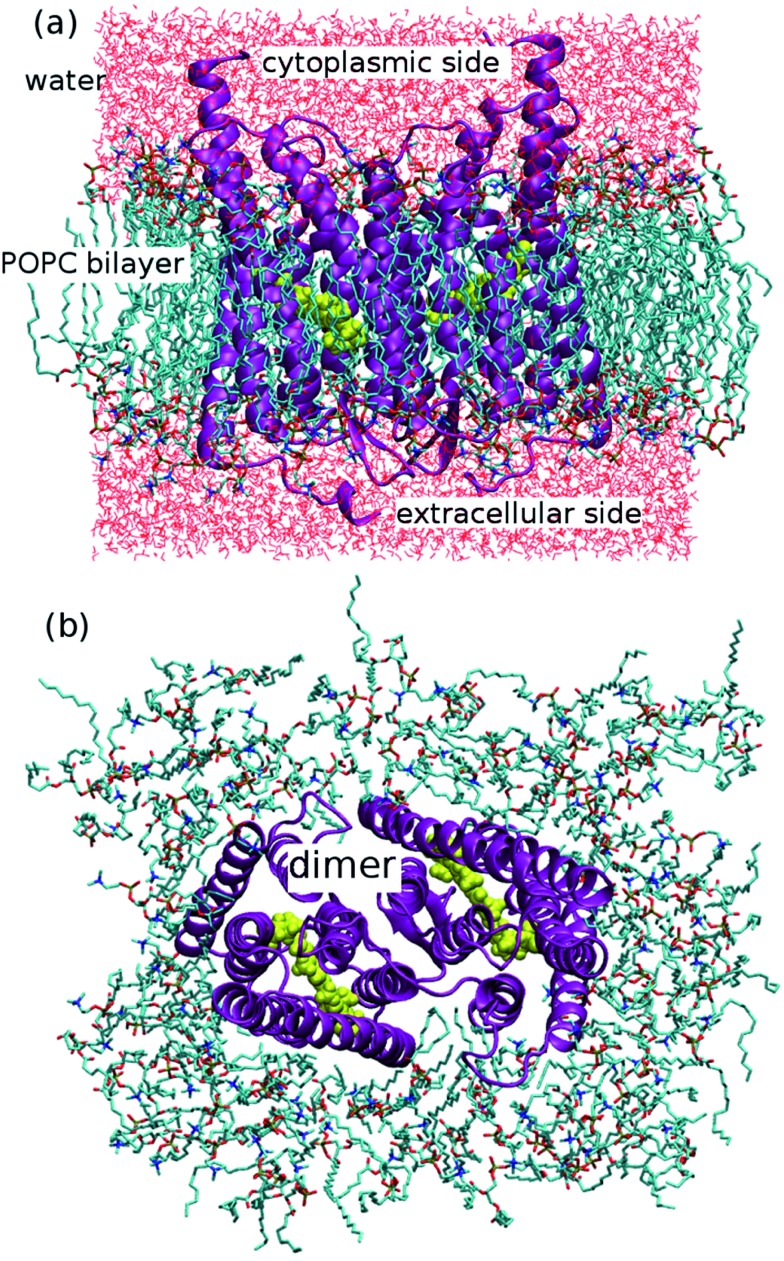
Model of ChR2: a protein dimer (purple) is inserted in a POPC lipid bilayer and surrounded by water molecules at both cytoplasmic and extracellular sides. The retinal chromophores (yellow) are covalently linked to K257 side chains. (a) Front view; (b) upper view, rotated by 90 degrees.

The ChR2 models developed in this study were built using the C1C2 crystal structure^17^ as starting template (all-*trans* retinal conformation). First, the ChR2-WT model was constructed as described in our previous study.^19^ The resulting monomer was used as initial structure to build a ChR2 dimer, surrounded by a POPC (1-palmitoyl-2-oleoylphosphatidylcholine) bilayer as lipid membrane, and water molecules as solvent (see Fig. 2). As previously proposed,^19^ apart from the water molecules resolved by X-ray crystallography, additional water molecules were placed inside the dimer by using the DOWSER program, which locates internal protein cavities through hydrophilicity calculations (*i.e.* through a calculation of the energy of interaction with the surrounding atoms).^39^

By this strategy, a water molecule was found bridging the C128 and D156 residues (the DC gate) in ChR2-WT, as proposed in previous studies.^19^,^33^

Standard protonation states were assumed, except for the E90^40^ and D156^17^,^19^ residues, both modeled as neutral. The RSBH^+^ potential counterions (D253 and E123 residues) were taken to be negatively charged, as deduced from previous QM/MM simulations^18^,^19^ and spectroscopic experiments.^13^,^19^–^22^

The ChR2-C128T model was built following the same procedure as for the ChR2-WT model, using the MMTSB tool set to implement the point mutation.^41^

The models were gradually equilibrated introducing selected restraints in order to preserve all ChR2 structural features, with special attention on the active site (see ESI† for details). A final 10 ns MM equilibration was performed for both models in a NPT-ensemble with a pressure of 1 bar and a temperature of 300 K.

For production purposes, we carried out QM/MM simulations, including in the QM region the retinal chromophore RSBH^+^ and the main residues constituting the active site: K257, E123, D253, T127 and K93 side chains, and the four water molecules found in the vicinity of the RSBH^+^ (see ESI† for details). QM/MM simulations are necessary since MM force fields are not able to describe the strongly hydrogen-bonded active sites of retinal proteins,^19^,^38^,^42^ a lethal failure especially when considering the prominent role of protein-bound water molecules in microbial rhodopsins.^43^ The selection of a large QM region was deemed necessary to obtain converged structures, as this had been crucial in previous rhodopsin studies focusing on the active site.^18^,^19^ Restraints were applied on the position of the oxygen atoms of the QM water molecules, in order to retain the QM description of the active site. Otherwise, QM and MM water molecules could interchange, leading to an unbalanced description and QM convergence problems.

Five QM/MM trajectories of 1 ns time were produced for each model, ChR2-WT and ChR2-C128T.

Considering the resonance Raman and IR spectroscopy studies which indicate the additional presence of the 13-*cis* retinal configuration in ChR2 dark state,^13^,^14^ we built the corresponding models for ChR2-WT and ChR2-C128T, adopting the same QM/MM setup (see ESI† for details). In both cases (all-*trans* and 13-*cis* retinal configurations) the RSBH^+^ has a similar position in the protein-retinal complex (Fig. S3c in the ESI†). In the present study, we calculated five QM/MM trajectories of 1 ns each for ChR2-WT and ChR2-C128T bound to 13-*cis* retinal.

In order to reproduce recent low temperature experiments,^16^ we calculated 14 QM/MM trajectories of 1 ns each for Chr2-WT bound to all-*trans* retinal. In each trajectory the temperature is decreased within the first 10 ps from 300 to 100 K and is kept at 100 K for the remaining time.

Moreover, the ground-state transition between different E123 side-chain conformations was studied through well-tempered metadynamics.^44^–^46^ A 100 ns classical MM simulation was performed for both ChR2-WT and ChR2-C128T bound with all-*trans* and 13-*cis* retinal (see ESI† for details).

For comparison purposes, a QM/MM trajectory of 1 ns time was performed for a BR model with the initial structure taken from our previous study.^9^ In this case, the QM region included the retinal chromophore (in all-*trans* configuration), the K219, D85 and D212 side chains, and the three water molecules forming the typical BR active site pentagonal cluster.

All MD simulations were performed with the GROMACS package,^47^ including metadynamics. The Charmm36 classical force field^48^ was applied, with appropriate parameters for the lipid membrane.^49^ The TIP3P model^50^ was used to describe water molecules. QM/MM trajectories were run applying the DFTB3/3OB (extended self-consistent-charge Density-Functional Tight-Binding method for biomolecules) method^51^–^53^ to the QM region, as recently implemented in the GROMACS package.^54^ DFTB3/3OB has been shown to model hydrogen-bonded networks with similar accuracy as full DFT calculations using medium sized basis sets,^51^ which is essential for a reliable modeling of the extended hydrogen-bonded structures in ChR2. Since trajectories on the ns time scale were required to study the formation and stability of hydrogen bonds, the application of DFTB/MM was preferred to the computationally more expensive DFT/MM. Indeed, DFTB/MM has been successfully applied to retinal proteins in the last years, in particular showing how infrared and absorption spectroscopy are sensitive to the active site structure.^33^,^34^,^38^

### Excited-state calculations

A highly accurate and extensive description of the active site by the DFTB3 method (in the framework of a QM/MM scheme) was considered essential to obtain reliable ground-state structures, followed by a calculation of the electronic excited-state energies. It had been shown previously that the remaining binding pocket (*i.e.* the other amino acids surrounding the retinal chromophore apart from E123 and D253) is responsible only for a minor hypsochromic (*i.e.* blue) shift when studying color tuning, and the influence of the rest of the protein is very minor.^9^ Therefore, the region outside the active site can be safely treated by classical force fields.

Two different strategies were applied in order to evaluate the absorption spectrum properties.

(i) First, 50 geometries randomly selected from the QM/MM trajectories of ChR2-WT were optimized at the DFTB3/Charmm36 level of theory with the CHARMM37b1 suite of programs^55^ to ensure that harmonic fluctuations of the structures around the energy minima are avoided. For each minimized structure, a single-point calculation of the electronic excitation energy was performed with the SORCI (Spectroscopy-Oriented multireference Configuration Interaction) method,^56^ as implemented in the ORCA program package.^57^ Moreover, the excitation energy of one BR minimized structure was calculated with the same SORCI setup, for comparison purposes. Indeed, since the BR active site has a well-defined ground state minimum, the geometry optimization of different trajectory snapshots leads to the same minimum energy structure, as previously discussed^58^ (see also discussion in the ESI†).

A complete active space of 12 electrons in 12 orbitals was selected, including the six π and six π* orbitals of the retinal chromophore. Three roots (the ground state and the two low-lying singlet excited states) were calculated by equal state averaging on each root (see the ESI† for details). The split-valence basis set def2-SV(P) was applied.^59^,^60^ All thresholds of the SORCI method were set in accordance with previous studies.^58^,^61^ A comparison with the established CASPT2 method^62^ has previously demonstrated that, for retinal proteins, SORCI is a reliable method to describe excitation energies.^61^

(ii) In order to evaluate dynamical effects on the absorption spectra of ChR2-WT and ChR2-C128T, the semi-empirical OM2/MRCI (Orthogonalization Model 2/Multi-Reference Configuration Interaction) method^63^,^64^ was applied on a statistically meaningful number of 20 000 QM/MM trajectory snapshots. For comparison, the same strategy was applied to the BR model, calculating the OM2/MRCI excitation energy of 1000 snapshots generated by the QM/MM trajectory. The OM2/MRCI method has been extensively tested^65^ and successfully applied to BR, sensory rhodopsin II, and bovine rhodopsin.^58^,^66^ The main advantage offered by OM2/MRCI, with respect to *ab initio* methods as SORCI and CASPT2, is the possibility to use a large active space (in this study: 20 electrons in 20 orbitals, including single and double excitations, see ESI† for details) at small computational cost. On the other hand, OM2/MRCI tends to overestimate the excitation energy in the case of retinals,^59^ and it is thus advisable to validate its use in the present case. Therefore, absorption energies were calculated with OM2/MRCI for the same 50 ChR2-WT geometries previously studied at the SORCI level. Different QM/MM setups were tested (see ESI†). We found that the SORCI excitation energies are well reproduced by applying a systematic hypsochromic shift of *ca.* 0.3 eV to the OM2/MRCI results obtained by only including the retinal chromophore in the QM region. In previous publications we have emphasized the importance of protein polarization effects in order to estimate both absolute and relative excitation energies.^38^,^67^–^71^ Protein polarization can be included using a polarizable force field (MMpol), where both the QM and MMpol treatments have to be solved iteratively. This is a very costly procedure, which is feasible only for static structures. Therefore, in the present work, we sample the trajectories only using standard fixed charge QM/MM techniques, which leads to a blue shift in the excitation energies of about 0.1–0.2 eV.^68^

All OM2/MRCI calculations were performed with the MNDO2005 program.^72^

## Results and discussion

### Active site structural motifs

The unrestrained QM/MM dynamics simulations show that both ChR2-WT and ChR2-C128T active sites (with all-*trans* or 13-*cis* retinal) are heterogeneous, which may be explained by: (i) the coexistence of three RSBH^+^ hydrogen-bonding patterns in a rather flexible environment, and/or (ii) the coexistence of two E123 side-chain conformations (Fig. 1b and c).

### RSBH^+^ hydrogen-bonding patterns

We adopted the following criteria to be fulfilled in order to assign a hydrogen bond: (i) angle between the formal proton H^+^, RSBH^+^ donor N, and acceptor A (–^+^HN···A–) smaller than 30°, and (ii) distance between N and A less than 3.5 Å. Three hydrogen-bonding patterns were found in ChR2-WT with all-*trans* retinal, indicating a direct interaction between the RSBH^+^ and the negatively charged side chain of E123 or D253 (Fig. 1b and c) or between the RSBH^+^ and a water molecule (Fig. 1d).

**Fig. 3:**
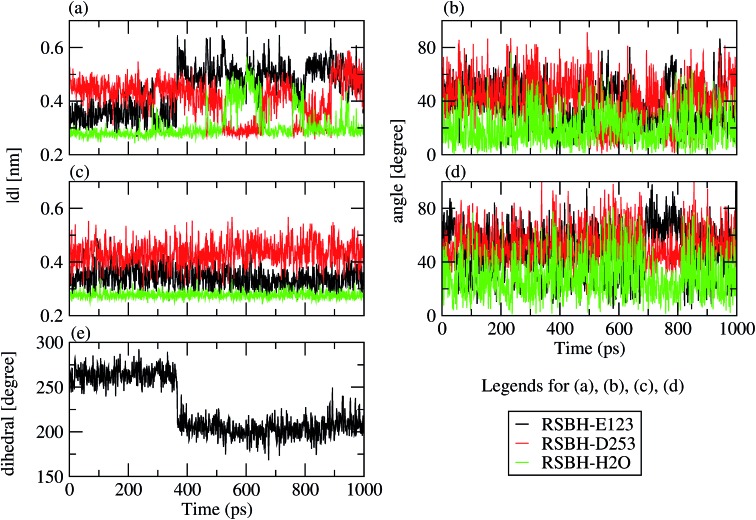
Stability of the hydrogen-bonding patterns (a–d) during the simulation time: the hydrogen-bond donor–acceptor distance of ChR2-WT (a) and BR (c); the formal H^+^–donor–acceptor angle of ChR-WT (b) and BR (d). Dihedral angle around the C_β_–C_γ_ bond (e) characterizing the E123 side-chain conformation: E123-upward/E123-downward for a dihedral angle larger/smaller than 240°.

Thus, the active site of ChR2 shows three distinct hydrogen-bonded motifs: –RSBH^+^···^–^O–(E123), –RSBH^+^···^–^O–(D253), and –RSBH^+^···OH_2_. Transitions between any two of the three hydrogen-bonding patterns could be observed in the trajectories (see movies in the ESI†). This is also evident from the temporal evolution of the distance and angle criteria for defining hydrogen bonds (see Fig. 3). Structures with donor–acceptor distances larger than 3.5 Å (criterion ii) were clustered due to the directionality of the RSBH^+^. The RSBH^+^···^–^O angle was compared to those of the three distinct motifs (see above) and assigned accordingly.

It should be highlighted that the protein bound with 13-*cis* retinal shows the same active site structural motifs as the protein all-*trans* retinal complex. This is understandable considering the very similar RSBH^+^ position of the two retinal configurations in the complexes (Fig. S3c in the ESI†).

Note that, according to the present simulations, –RSBH^+^···^–^O–(E123), –RSBH^+^···^–^O–(D253) and –RSBH^+^···OH_2_ patterns represent 38.6, 31.8 and 29.6%, respectively, of the 10 ns simulation time, for both retinal configurations. Hence, within the limits of our approach, we can conclude that E123, D253 and H_2_O could each be the potential hydrogen-bonding partner of RSBH^+^.

A comparison with BR is informative, especially with regard to the water arrangement within the active site: light-adapted BR contains three water molecules forming a pentagonal cluster with the D85 and D212 side chains, with one water molecule involved in a stable hydrogen bond with the RSBH^+^ (Fig. 1e).^73^ Indeed, our QM/MM simulation shows that this setting is well preserved (Fig. 3c and d), thus reproducing this characteristic BR structural motif. On the other hand, in the case of ChR2-WT, all three identified structural motifs feature two water molecules bridging the E123 and D253 side chains (Fig. 1b–d), and two additional water molecules are involved (*i.e.* four in total) when switching to the –RSBH^+^···OH_2_ pattern (Fig. 1d). We call these arrangements ChR2 half-barrel (when involving two water molecules) and ChR2 barrel (when involving four water molecules), in contrast to the BR pentagonal cluster.

The same structural motifs were found in ChR2-C128T with both retinal configurations. However in this case, in contrast to ChR2-WT, the RSBH^+^···OH_2_ motif occupies 72.1% of the 10 ns simulation time and dominates over the –RSBH^+^···^–^O–(E123) (4.5%) and –RSBH^+^···^–^O–(D253) (23.4%) motifs, indicating that a water molecule could play the role of prominent counterion. The ChR2 barrel and ChR2 half-barrel water clusters were also sampled in ChR2-C128T.

Therefore, ChR2-WT and ChR2-C128T seem to have the same RSBH^+^ hydrogen-bonding patterns, independent of the retinal conformation. ChR2-WT has a preference for –RSBH^+^···^–^O–(E123), ChR2-C128T for –RSBH^+^···OH_2_. This could be one structural reason for the different fine structure of the absorption spectra of ChR2-WT and ChR2-C128T.^25^

### E123 side-chain conformation

**Fig. 4:**
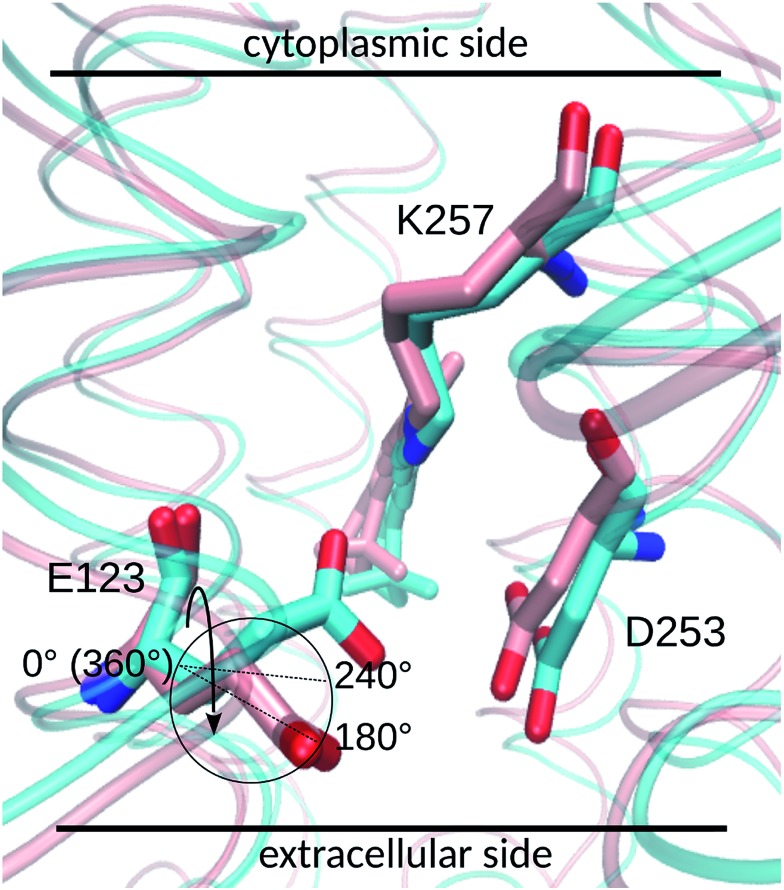
The upward (cyan backbone) and downward (pink backbone) E123 side-chain conformations. The cycle highlights the reaction coordinate, *i.e*. the dihedral angle around the C_β_–C_γ_ bond (depicted by the arrow), for the well-tempered metadynamics calculation. When the dihedral angle is larger (smaller) than 240°, the E123 side chain is in an upward (downward) conformation.

Two E123 side-chain orientations were sampled for both ChR2-WT and ChR2-C128T: (a) towards the cytoplasmic side (E123-upward), or (b) towards the extracellular side (E123-downward) (Fig. 4). These differences influence the RSBH^+^ hydrogen-bonding patterns as shown in Fig. 3, where the E123 side-chain conformation is characterized by the dihedral angle around the C_β_–C_γ_ bond (upward/downward for an angle above/below 240°). In more detail, in the case of E123-upward, the –RSBH^+^···^–^O–(E123) or the –RSBH^+^···OH_2_ motif is dominant; in the case of E123-downward, the –RSBH^+^···OH_2_ or the –RSBH^+^···^–^O–(D253) motif is preferred.

**Fig. 5:**
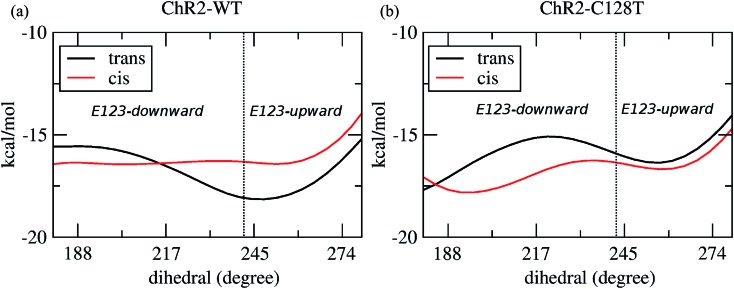
The free energy as a function of the dihedral angle around the C_β_–C_γ_ bond of E123. (a) ChR2-WT bound with all-*trans* retinal (black line), and bound with 13-*cis* retinal (red line). The dihedral angle ranges corresponding to E123-upward and E123-downward are separated by a dotted line. (b) ChR2-C128T bound with all-*trans* retinal (black line), and bound with 13-*cis* retinal (red line). The dihedral angle separating range is the same as in (a).

In order to evaluate our sampling, we calculated at the MM level free energy profiles for ground-state transitions between the two E123 conformations (see Methods and ESI† for details). The profiles for ChR2-WT and ChR2-C128T are shown in Fig. 5. We can see that, in ChR2-WT bound with all-*trans* retinal (Fig. 5a, black line), there is an *ca.* 2.5 kcal mol^–1^ rise in free energy from E123-upward to E123-downward, while the reverse path is downhill. While bound with 13-*cis* retinal (red line), there is almost no free energy barrier from E123-upward to E123-downward or *vice versa*. These results are in good agreement with our QM/MM simulations, which sampled more E123-upward conformations (63%) than E123-downward conformations, with dihedral angles around the C_β_–C_γ_ bond ranging from 180 to 280° in the QM/MM simulations.

In ChR2-C128T bound with all-*trans* retinal (Fig. 5b, black line), the calculated free energy barrier from E123-upward to E123-downward is *ca.* 1.2 kcal mol^–1^, while it is *ca.* two times higher for the reverse transition. When bound with 13-*cis* retinal (red line), the barrier is lower compared with the all-*trans* retinal bound complex, but it is still *ca.* two times higher from E123-downward to E123-upward than for the reverse transition. This is quite consistent with our QM/MM simulations, where *ca.* 92% of the sampled structures correspond to the E123-downward conformation.

In conclusion, the ChR2-WT and ChR2-C128T active sites are quite heterogeneous compared with the rigid BR active site. The flexible water molecules and E123 side-chain conformations result in three RSBH^+^ hydrogen-bonding patterns, which might contribute to the characteristic absorption spectrum of ChR2. Such high flexibility determines that both E123 and D253 side chains are potential RSBH^+^ proton acceptors.

### Active site at low temperature

In the trajectories at 100 K the same structural motifs (the three hydrogen-bonding patterns and the two E123 side-chain conformations (Fig. 1b and c) as in the trajectories at 300 K are observed. However, the trajectories at 100 K show nearly no transitions between the different structural motifs, corresponding to a rigid structure. Indeed, transitions between two structural motifs are observed only in 2 out of 14 trajectories, while at 300 K several transitions per nanosecond are seen.

Comparing the starting structures (at 300 K, before cooling) with the resulting structures at 100 K, the –RSBH^+^···^–^O–(E123) structural motif is found in higher amount at 100 K (Table S2 in ESI†). Therefore, significant structural changes are observed while cooling, finally favoring the –RSBH^+^···^–^O–(E123) pattern. This could be expected since the –RSBH^+^···^–^O–(E123) pattern is the most abundant at 300 K, indicating a slightly lower free energy (*ca.* 2 kcal mol^–1^) of this conformation.

### Absorption spectrum

**Fig. 6:**
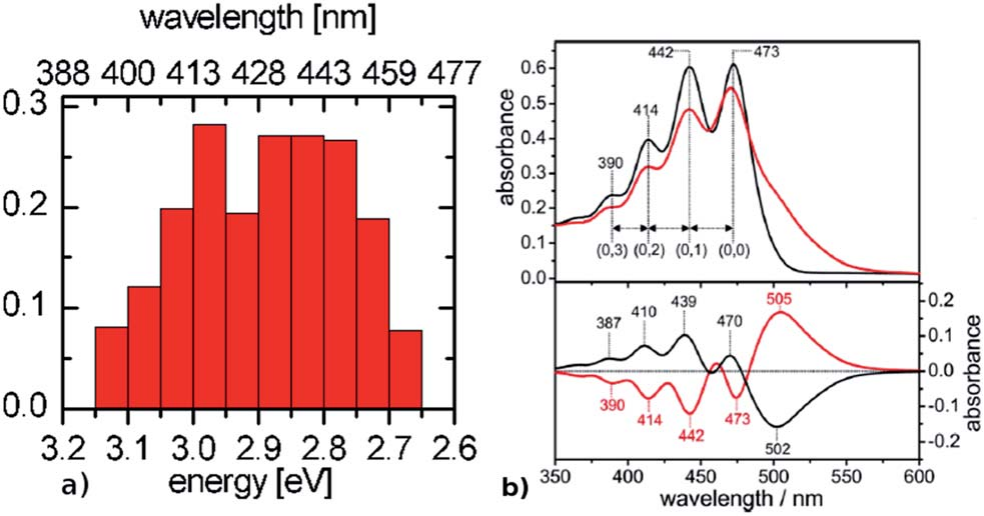
Comparison between (a) simulated ChR2-WT and (b) ChR2-H134R experimental absorption spectrum at 4 K (upper panel, black: before illumination at 455 nm, red: after illumination at 455 nm), with its fine structure resolved in the second derivative spectrum (lower panel, adapted from Bruun *et al*.^15^). The histogram is based on SORCI calculations at optimized QM/MM structures (see text).

The spectra of BR and ChR2 differ mainly in the wavelength of maximum absorbance and the number of peaks: the absorption spectrum of light-adapted BR with exclusively all-*trans* retinal chromophore is constituted by a single peak centered at around 570 nm (2.17 eV),^74^,^75^ while in ChR2 dark-adapted for several minutes (DAapp) – with a roughly 70 : 30 mixture of all-*trans* to 13-*cis* – it is characterized by three major peaks at 473 nm (2.62 eV), 442 nm (2.80 eV) and 414 nm (2.99 eV), resolved by calculating the second derivative spectra^15^ (see Fig. 6). Therefore, when considering the experimental results, both types of rhodopsin do absorb in the visible range, with a considerable hypsochromic shift in the case of ChR2.

As discussed in the preceding section, both ChR2-WT and ChR2-C128T may have active site structural motifs of –RSBH^+^···^–^O–(E123) or –RSBH^+^···^–^O–(D253), in which the anionic side chains of E123 and D253 (RSBH^+^···^–^OOC) could stabilize the electronic ground state S_0_ and enlarge the electronic energy gap with respect to BR (S_0_–S_1_). This has been suggested to be the origin of the detected hypsochromic shift between ChR2 and BR.^17^ A detailed QM/MM analysis, however, has shown that the overall electrostatic interaction with the protein environment is responsible for a large part of the shift, while the contribution of the hydrogen-bonding network is moderate.^9^

The presence of a single peak in the absorption spectrum of BR was explained by a single active site structure (*i.e.* the pentagonal cluster) representing the global ground-state minimum, with harmonic oscillations due to thermal effects leading to homogeneous spectral broadening.^34^ On the other hand, we have shown in the previous section (see Fig. 3) that ChR2 exhibits structural disorder and can adopt different but more defined ground-state structural motifs, which could explain the inhomogeneous broadening observed in its absorption spectrum (Fig. 6b). We calculated the electronic excitation energy of 50 ChR2-WT geometries with the SORCI method. Each geometry corresponds to a random snapshot taken from the QM/MM trajectories and optimized at the QM/MM level. As shown in Fig. 6a, the minimized geometries do not collapse to a global ground-state energy minimum, but basically maintain the initial structural motif (see ESI†), and thus correspond to a multitude of local ground-state energy minima. This gives rise to a typical inhomogeneous spectral broadening: SORCI calculations at these geometries yield a wide distribution of excitation energies; the resulting absorption spectrum is centered between 2.85 and 3.00 eV and is 0.5 eV broad. On the other hand, we also calculated by SORCI the excitation energy of an optimized QM/MM snapshot of BR, being representative of the wavelength at maximum absorbance. The value of 2.35 eV indicates a bathochromic (*i.e.* red) shift of *ca.* 0.50 eV with respect to ChR2-WT, in good agreement with the experimental shift of 0.45 eV.

When compared to the experimental absorption spectra, the SORCI-based spectra for both the ChR2-WT and BR models exhibit a hypsochromic shift of *ca.* 0.25 eV, which can be explained in terms of the applied methodology: the electric field generated by the classical MM point charges surrounding the QM region tends to be overestimated; it could be (at least partially) moderated by using a polarizable force field, especially considering the presence of ionized groups in the protein interior surrounding the active site.^76^ In the case of BR a bathochromic shift between 0.2 and 0.3 eV is expected when including polarization effects.^61^,^77^,^78^ Nevertheless, the considerably higher computational cost required by polarizable force fields compared to classical ones makes this option unpractical for the present study, which is focused on extensive simulations of the different ChR2 structural motifs.

**Fig. 7:**
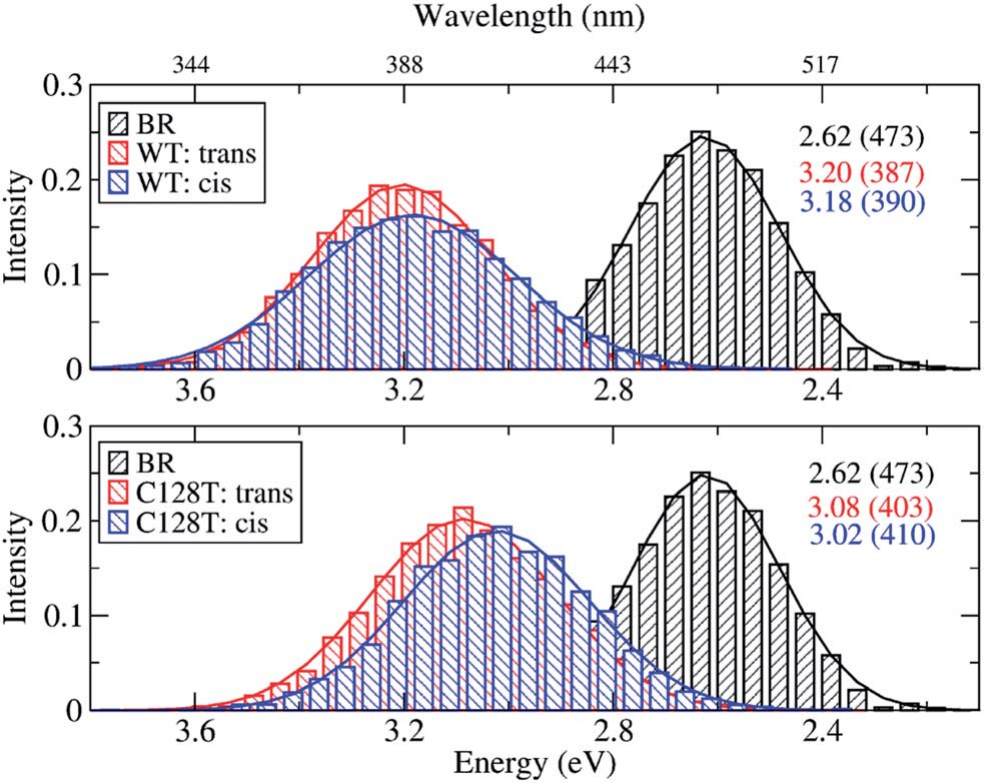
Comparison between simulated absorption spectra of ChR2-WT, ChR2-C128T and BR. The histograms are based on OM2/MRCI calculations of snapshot geometries from QM/MM trajectories. Gaussian functions are used to convolute the spectra and the corresponding maxima are listed in the same color in eV (nm). The fitted parameters are listed in Table S3.† Notation: “*trans*” refers to snapshots bound with all-*trans* retinal; “*cis*” refers to snapshots bound with 13-*cis* retinal.

Because of the inherent flexibility shown by the ChR2-WT active site, an exhaustive ground-state structural sampling was considered necessary in order to properly describe dynamical effects on the absorption spectrum of ChR2 and, for comparison, BR. Fig. 7 shows the results obtained from OM2/MRCI calculations on 10 000 snapshots for ChR2-WT and for ChR2-C128T (5000 snapshots of all-*trans* retinal, and 5000 of 13-*cis* retinal) extracted from QM/MM simulations. The results for BR from 1000 snapshots are also shown. During data analysis we should keep in mind that a hypsochromic shift of *ca.* 0.3 eV has to be considered when passing from SORCI to OM2/MRCI.

The BR active site is responsible for a consistent bathochromic shift of 0.58 eV with respect to ChR2-WT bound only with all-*trans* retinal. This result is quite close to that of the experiment (0.45 eV) within the limits of the OM2/MRCI accuracy. In the case of 13-*cis* retinal, the shift of 0.56 eV is almost the same as that of all-*trans* retinal. This indicates that both retinal configurations could equally be responsible of the absorption spectral shape.

In ChR2-WT and ChR2-C128T, the computed spectra for the all-*trans* retinal and the 13-*cis* retinal bound complexes have very similar absorption maxima, resulting in a hypsochromic shift of 0.45 eV with respect to BR. Hence, the assumption that the two retinal configurations coexist cannot be verified by examining the absorption spectrum of ChR2.

**Fig. 8:**
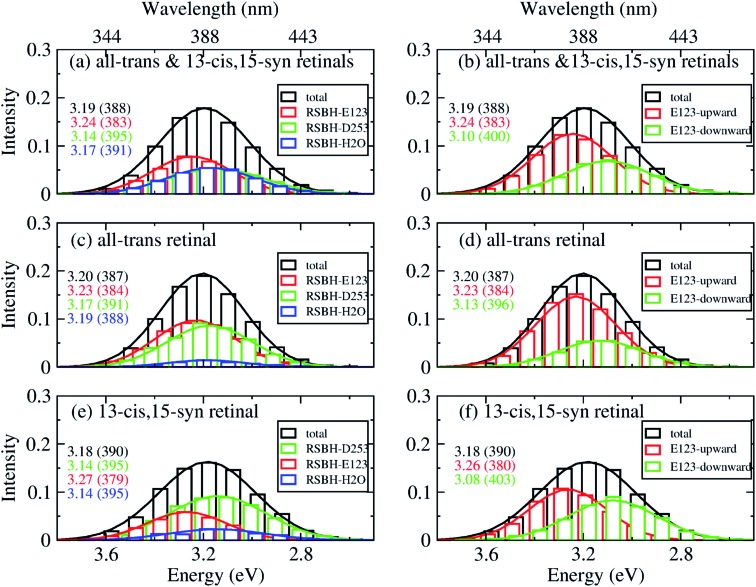
A deconvolution based on the assignment of structural motifs is proposed for ChR2-WT with mixed all-*trans* and 13-*cis* retinals (a and b), with all-*trans* retinal (c and d), and with 13-*cis* retinals (e and f). The contributions of the three RSBH^+^ hydrogen-bonding patterns (a, c and e) and the two E123 side-chain conformations (b, d and f) are depicted. The histograms are based on OM2/MRCI calculations of snapshot geometries from QM/MM trajectories. Gaussian functions are used to convolute the spectra and the corresponding maxima are listed in the same color in eV (nm). The fitted parameters are listed in Table S3.† Here, “total” denotes the histogram from all the snapshots; “RSBH-E123” corresponds to the –RSBH^+^···^–^O–(E123) hydrogen-bonding pattern; “RSBH–D253” corresponds to the –RSBH^+^···^–^O–(D253) hydrogen-bonding pattern; “RSBH–H_2_O” corresponds to the –RSBH^+^···^–^OH_2_ hydrogen-bonding pattern; “E123-upward” and “E123-downward” refer to snapshots with the E123 side chain upwards and downwards, respectively.

**Fig. 9:**
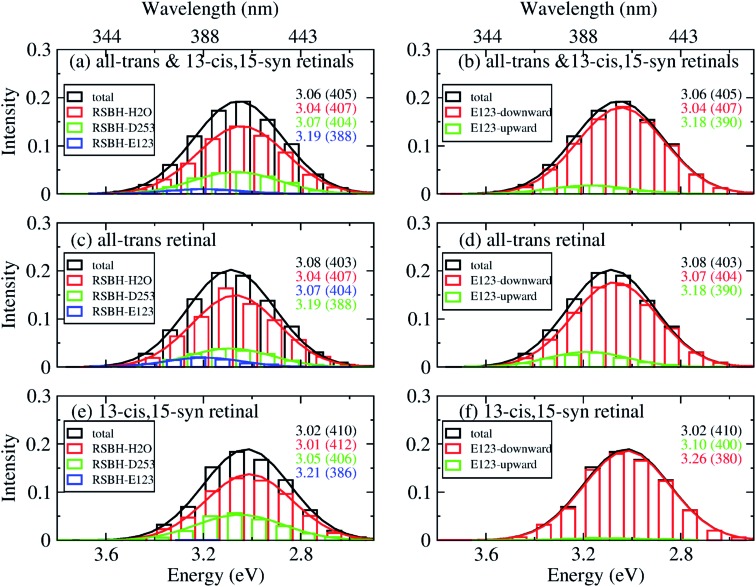
A deconvolution based on the assignment of structural motifs is proposed for ChR2-C128T, applying the same methodology and notations as in Fig. 8.

In order to gain insight into the composition of the spectra, we deconvoluted the overall histograms of the all-*trans* retinal bound protein, of the 13-*cis* retinal bound protein, and of the mixed all-*trans* and 13-*cis* retinals bound protein, to analyze the influence of the different active site structural motifs in ChR2-WT (Fig. 8) and ChR2-C128T (Fig. 9), in terms of the contributions of the three RSBH^+^ hydrogen-bonding patterns and the two E123 side-chain conformations discussed in the previous section.

In ChR2-WT bound with mixed all-*trans* and 13-*cis* retinal (Fig. 8a), the –RSBH^+^···^–^O–(D253) pattern contributes to the long wavelength part of the absorption spectrum. A maximum hypsochromic shift of 0.10 eV is identified between the –RSBH^+^···^–^O–(E123) and –RSBH^+^···^–^O–(D253) patterns, whereas the maximum shift is 0.07 eV between the –RSBH^+^···^–^OH_2_ and –RSBH^+^···^–^O–(D253) patterns. Experimentally, the two shoulders of the fine structure of the absorption spectrum are 0.18 and 0.37 eV, respectively, hypsochromically shifted with respect to the absorption maximum at long wavelength. Our calculated results are comparable to the experiment taking into account the accuracy of the applied methodology. Thus, in the complex bound with mixed retinals, the three hydrogen-bonding patterns might be the structural origin of the fine structure in the absorption spectrum of ChR2-WT.

In the case of the all-*trans* retinal complex (Fig. 8c), a hypsochromic shift of 0.06 eV is observed between the –RSBH^+^···^–^O–(E123) and –RSBH^+^···^–^O–(D253) patterns; while a narrower hypsochromic shift of 0.02 eV is found between the –RSBH^+^···^–^OH_2_ and –RSBH^+^···^–^O–(D253) patterns.

In the case of the 13-*cis* retinal complex (Fig. 8e), the –RSBH^+^···^–^O–(D253) and the –RSBH^+^···^–^OH_2_ patterns have the same absorption maximum, relatively, the maximum of the RSBH^+^···^–^O–(E123) pattern is 0.13 eV hypsochromically shifted.

From the perspective of the E123 side-chain conformation, E123-upward shows a hypsochromic shift of 0.10 eV, 0.18 eV, and 0.14 eV with respect to E123-downward in the pure all-*trans* retinal bound protein, the 13-*cis* retinal bound protein, and the mixed-retinal bound protein (Fig. 8b, d and f), in good agreement with the experimental value (0.18 eV). This suggests that the E123 side-chain conformation may also be the key structural reason for the fine structure in the absorption spectrum of ChR2-WT.

In ChR2-C128T, the –RSBH^+^···OH_2_ and the –RSBH^+^···^–^O–(D253) patterns give almost the same absorption maximum, and are responsible for the long wavelength of the absorption spectrum regardless of the retinal configuration (Fig. 9a, c and e). The –RSBH^+^···^–^O–(E123) pattern gives rise to a hypsochromic shift of *ca.* 0.15 eV with respect to the –RSBH^+^···^–^O–(D253) or –RSBH^+^···OH_2_ pattern. On the other hand, there is a difference of *ca.* 0.15 eV between the E123-upward maximum and the E123-downward maximum. Therefore, the RSBH^+^ hydrogen-bonding patterns and the E123 side-chain conformation should both be considered as structural features influencing the absorption properties of ChR2-C128T.

## Conclusions

Our QM/MM simulations provide novel insights into the active site of ChR2, a protein of considerable interest due to its important role in optogenetic applications. Up to now, the only available structure is the X-ray structure of the C1C2 chimera, which only contains one water molecule in the active site and may have a different active site structure from ChR2.

The detailed characterization of the active site in channelrhodopsins is of great importance not only to rationalize color tuning studies, but also for the following reasons: the active site is in direct connection with the central gate, and activation of the ion conductance is intimately connected with the active site. Moreover this connection is also critical for the ion channel selectivity. The central gate and its connection with the active site has been recently altered to convert the ion selectivity from cation to anion selectivity. A major interest of the neuroscientists is to convert the ion selectivity to a K^+^ or Ca^2+^ selectivity, which is unsuccessful so far. For such an engineering we need more detailed knowledge about the active site, central gate and inner gate, and about water content and proton-networks in particular.

Our simulations suggest a heterogeneous active site structure, having several local minima with small barriers being sampled at room temperature on a nanosecond timescale. This energy landscape is sensitive to mutations, as shown for C128T and may lead to interesting temperature dependence of the active site structure, especially when approaching lower temperatures.

At 100 K we obtain hydrogen-bonding patterns that are much less flexible than those at 300 K. A rigid active site structure is obtained, with one hydrogen-bonding pattern clearly favored which is the RSBH^+^···^–^O–(E123) motif. In this case, the RSBH^+^–^–^OOC(E123) distance is smaller than in BR, which is consistent with the finding of a previous solid-state NMR study^16^ (for details see ESI, Table S3†). Please note that at 300 K the RSBH^+^–^–^O distance results from an average over all three hydrogen-bonding patterns, leading to a larger distance. This nicely shows the importance of temperature for the active site structure, as shown recently also for BR.^38^

The heterogenous active site explains the multi-peak nature of the absorption spectrum of ChR2-WT, which is independent of the retinal configuration: not only the different E123 side-chain conformations, but also the distinct RSBH^+^ hydrogen-bonding patterns contribute to the fine structure of the final spectrum. The –RSBH^+^···^–^O–(D253) and the –RSBH^+^···OH_2_ patterns contribute to the long wavelength of the absorption spectrum, while the –RSBH^+^···^–^O–(E123) pattern is responsible to the sub-peak of the spectrum. This conclusion is also feasible for ChR2-C128T. However, in ChR2-C128T, only *ca.* 5% simulations sampled the –RSBH^+^···^–^O–(E123) pattern; in ChR2-WT, the –RSBH^+^···^–^O–(E123) pattern occupies *ca.* 38% of the simulation time, which is correlated to the E123 side-chain conformations as discussed previously. This difference might be the structural reason explaining the fine structure difference of the spectrum between ChR2-WT and ChR2-C128T.

The applied computational methodology has been used frequently in recent years to compute absorption energies for various retinal proteins with a good success rate. This suggests that the effect of different retinal conformations and protein structures on computed excited states energies is well reproduced. Since the relative excitation energies of all-*trans* retinal in BR, ChR2-WT and ChR2-C128T agree quite well with experiment, we conclude that the structural model presented here represents the ChR2 structure probed in experiment with sufficient accuracy.

On the other hand, the *cis*-retinal conformers show the same structural motifs as the *trans*-retinal conformers; within the computational model, the *cis*-retinal does not change the absorption spectrum with respect to the *trans*-retinal conformers. This implies that we cannot exclude the 13-*cis* retinal from the dark-state ChR2 on computational grounds, however, this point has now been clarified experimentally.^16^ In the current paper we present a study of a very flexible active site using quantum mechanical methods. Judging from the relative occupations, the flexible active site indicates free energy differences within a few kcal mol^–1^ between the different hydrogen-bonding patterns. As a consequence, very small inaccuracies in the quantum chemical descriptions can shift the occupations of the individual states as well as the temperature dependence. The required accuracy is definitely higher than the chemical accuracy of 1 kcal mol^–1^, which is hard to achieve for standard electronic structure methods. Therefore, the picture we present here has a qualitative meaning, rather than quantitative. Such highly flexible active sites pose a clear challenge to computational methods, and the results should not be over-interpreted.

Finally we note that, in order to answer the question concerning which (E123 and/or D253) is the proton acceptor, further investigation is needed. Especially, the different possible pathways should be carefully taken into account, and it constitutes an ongoing work of the authors. However, the flexibility of the active site shown here suggests that both residues are potential proton acceptors.

The present study can help in understanding the different and sometimes contradictory experimental results, since previously proposed –RSBH^+^···OH_2_, –RSBH^+^···^–^O–(E123) and –RSBH^+^···^–^O–(D253) patterns are found to dynamically coexist in the ChR2 active site. This may pave the way for a coherent and accurate description of the first steps in the ChR2 photocycle, including photoisomerization and later intermediates, which are at present largely unknown. The active site models presented here provide the necessary structural details to assess the role of retinal counterions in the formation of ChR2 hydrogen-bonding patterns, compared to BR and C1C2.

## Author contributions

The manuscript was written through contributions of all authors. All authors have given approval to the final version of the manuscript.

## Conflict of interest

The authors declare no competing financial interest.

## Supplementary Material

Supplementary information

Supplementary movie

Supplementary movie

Supplementary movie
