# Supplementary material for: Active site structure and absorption spectrum of channelrhodopsin-2 wild-type and C128T mutant
Source: Chem Sci. 2016 Feb 26;7(6):3879–91. doi: 10.1039/c6sc00468g (PMC6013792; doi:10.1039/c6sc00468g)
Supplement: Supplementary file 1 [file SC-007-C6SC00468G-s001.pdf]

Electronic Supplementary Material (ESI) for Chemical Science.
This journal is © The Royal Society of Chemistry 2016

Electronic Supplementary Information

Active Site Structure and Absorption Spectrum of Channelrhodopsin-2 Wild-Type and C128T Mutant

Yanan Guo,[†] Franziska E. Beyle,[†] Beatrix M. Bold,[†] Hiroshi C. Watanabe[¶], Axel Koslowski,[‡] Walter Thiel,[‡] Peter Hegemann,[‖] Marco Marazzi,*[†] and Marcus Elstner*[†]

[†] Department of Theoretical Chemical Biology, Institute of Physical Chemistry, KIT, Kaiserstrasse 12, 76131 Karlsruhe, Germany

[¶] Research Center for Advanced Science and Technology, The University of Tokyo, 4-6-1 Komaba, Meguro-ku, Tokyo 153-8904 Japan

[‡] Max-Planck-Institut für Kohlenforschung, Kaiser-Wilhelm-Platz 1, 45470 Mülheim an der Ruhr, Germany

[‖] Institute of Biology, Experimental Biophysics, Humboldt-Universität, Invalidenstraße 42, D-10115 Berlin, Germany

Content

1. Modelling ChR2: MM Equilibration
2. DC/DT gate
3. Modelling ChR2: QM/MM Production Run
4. SORCI vs OM2/MRCI: Excitation Energies
5. Cartesian Coordinates in Ångström of Representative QM/MM Optimized BR and ChR2 Active Site Structures (QM region)
6. Metadynamics
7. Gaussian Fit Parameters
8. References

1. Modelling ChR2: MM Equilibration

As described in the main text, ChR2-WT and ChR2-C128T models were built using a homology model of the C1C2 chimera X-ray structure (PDB code: 3UG9) as template. From there, we set up the respective protein dimers surrounded by a POPC membrane and water molecules as solvent. The complete model is constituted by 492 amino acids (246 amino acids per monomer), 107 POPC molecules, and 6882 water molecules (6880 water molecules in ChR2-C128T, since no water molecule is expected in the DC gate of each monomer, see main text), in a box of 8.22 nm × 6.74 nm × 7.45 nm.

After setting the protonation state of the protein residues (see main text), the structure of the active site was modeled taking into account previous spectroscopic and QM/MM dynamics simulations (see refs. [19-22] and references therein). More in detail, both E123 and D253 are deprotonated, and the side chain of E123 forms a hydrogen bond with the side chain of T127, allowing a water molecule to stay next to the $RSBH^+$ (Figure 1S).

The first step was the minimization of the potential energy. This was achieved by calculating 1000 steps with the steepest-descent method (tolerance of 1000 kJ mol^{-1} nm^{-1}). The next step was the equilibration of the solvent, which was achieved by simulating a NVT-ensemble for 10 ps at a temperature of 300 K, applying the Berendsen thermostat [1S] (time step: 2 fs). The heavy atoms of the protein and lipid membrane were restrained to their initial positions by harmonic potentials (force constant: 1000 kJ mol^{-1} nm^{-1}). Subsequently both (wt and C218T) systems (including the protein dimer and the lipid bilayer) were equilibrated for 10 ns in the NPT-ensemble, at a pressure of 1 bar (Parrinello–Rahman barostat [2S]) and a temperature of 300 K (Nosé–Hoover thermostat [3S,4S]). The time step was set to 2 fs, using the H-bond scheme for constraints as implemented in the LINCS algorithm within the GROMACS package.

As described in the main text, MM force fields show severe shortcomings in the description of strongly hydrogen bonded networks. This has been demonstrated in particular for the BR active site. Using standard force fields, the crystal water binding to the Schiff-base NH group is lost and leaves the active site. A similar finding has been reported for ChR in our previous papers (see refs. [18,19]). If this water is lost during MM equilibration, it will not be restored in QM/MM simulations due to the limited simulation time. Therefore, to avoid a bias due to the above described 10 ns MM equilibration, we harmonically restrained the positions of the oxygen atom of the water molecule and of the nitrogen and hydrogen atoms of the $RSBH^+$ were with a force constant of 10000 kJ mol^{-1} nm^{-1} during the whole MM equilibration procedure. Moreover, the hydrogen bond between the hydrogen atom of T127 and the oxygen atom of E123 was constrained to a length of 0.18 nm. Note that we manually rotated the side chain of E123 and the water molecule for each monomer, therefore, the two monomers are not guaranteed to have exactly the same starting active site structure. This further results in two starting points for the production QM/MD simulations as shown in Figure 1S. The Charmm36 force field was applied

in the framework of the GROMACS software package.

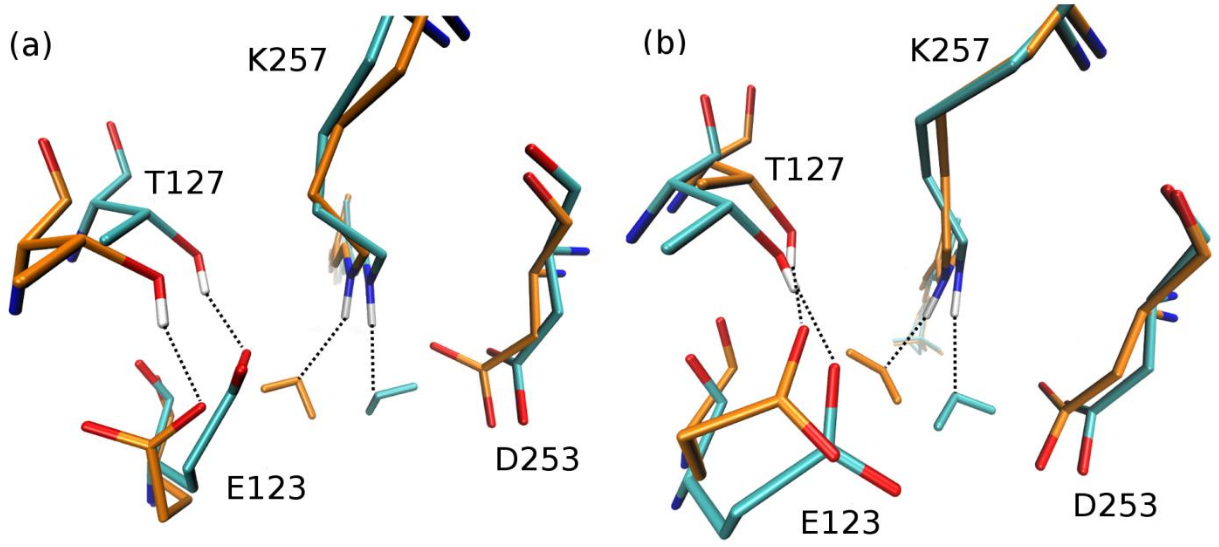

Figure 1S. Initial conditions selected for MM equilibration of the proposed ChR2 models: hydrogen bonds are set between E123 and T127 side chains, and between a water molecule (H_2O) and the protonated Schiff base, $RSBH^+$ (Ret: retinal). (a) ChR2-WT, (b) ChR2-C128T. The two structures are shown in backbone colors of cyan and orange, respectively.

2. The DC/DT Gate Structures

For ChR2-WT, the MM equilibration simulations show two different DC gate structures: (i) one water molecule bridges D156 and C128 (water bridged DC gate, Figure 2S (a)); (ii) D156 and C128 form a direct hydrogen bond (direct DC gate, Figure 2S (b)), where D156 serves as a donor and C128 as an acceptor. In the starting structures, both monomers contain the water as shown in Figure 2S,a. During equilibration, however, the water in monomer 2 is lost, while it stays in monomer 1. The water bridged DC gate has been proposed by previous classical MD simulations [18] and validated by QM/MM vibrational frequency calculations [33]. To further check this assignment for the present models, we calculated the stretching vibrational frequency of D156 side chain (*v*COO) for the above two DC gate structures using the normal mode analysis (NMA) with the CHARMM37b1 program at the DFTB3/MM level of theory, which has been validated in detail in ref [33]. The residues C128, D156, and the bridging water molecules (for water bridged DC gate) were included in the QM region, and the rest was treated as MM part. The calculated *v*COO value is 1730 cm^{-1} for the water bridged DC gate structure, which agrees well with the experimental data (1735 cm^{-1}) [5S], and 1693 cm^{-1} for the direct DC gate structure, i.e. 42 cm^{-1} red shifted compared with the FTIR value. We take this as a strong indication for the presence of one water molecule in the DC gate. The DC gate structure is strongly coupled to the active site, and the active site structures thus differ for the water bridged and direct DC gate models. Therefore, since a missing water in the DC gate may lead to wrong active site structures,

we discard the simulations of the monomer without DC water in the further analysis with QM/MM simulations.

To summarize, our findings in principle suggest that it is crucial to include the DC gate into the QM description since, in the MM description, the DC gate motif is not stable in ChR, and the bridging water tends to leave. This, however, happens during the extended MM equilibration step, which unfortunately cannot be performed using QM/MM, due to the long simulation times. Therefore, based on MM equilibration we had to discard trajectories where the water leaves the DC gate.

Regarding the QM/MM simulations, as long as the DC gate structure is intact, the DC gate does not have to be included into the QM zone because it is far away from the counterion region. If the DC gate is broken, however, the active site structure is severely impaired. Therefore, we monitored the DC gate during the QM/MM simulations. Including the DC gate into the QM zone would increase the simulation times significantly. However, we would like to note that all calculations were performed in a QM/MM environment, therefore the Coulomb effect (which is mainly responsible for the separation of excited states) is always taken into account.

For ChR2-C128T, the simulations sample a single direct hydrogen bond between T128 and D156 (Figure 2S (c)), as discussed in a previous theoretical study [33].

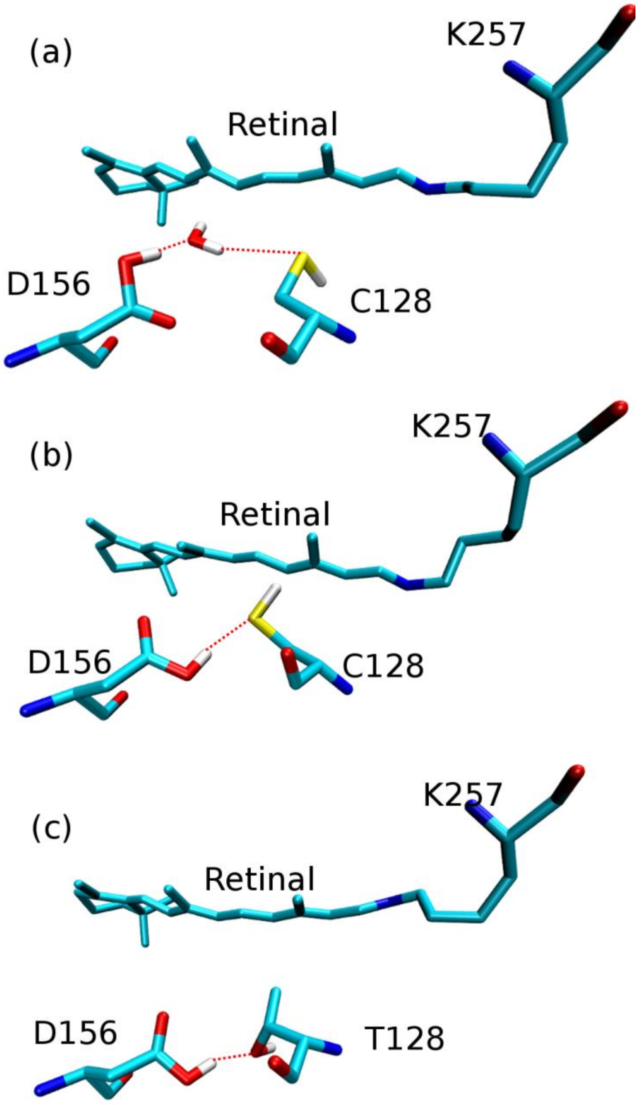

Figure 2S. The DC (D156-C128)/DT (D156-T128) gate structure sampled in the present simulations. (a) water bridged DC gate of ChR2-WT; (b) direct DC gate of ChR2-WT; (c) direct DT gate of ChR2-C128T.

3. Modelling ChR2: QM/MM Production Run

After MM equilibration, we performed QM/MM MD simulations with the GROMACS package using DFTB3 for QM region and the Charmm36 classical force field for the remainder of the system. The QM region (S-QM) includes the retinal, Lys257, the E123, D253, and K93 side chains, and four (three for ChR2-C128T) water molecules involved in the active site hydrogen bonding patterns. Four hydrogen link atoms are needed to treat the QM/MM boundary regions where a covalent bond is involved, *i.e.* between α- and β-carbon atoms of E123, D253, K257 and K93 residues.

The restraints imposed on the $active$ site during the MM equilibration were gradually removed

when including the active site in the QM region of the DFTB3/Charmm36 model, as summarized in Table 1S. Weak restraints on the QM waters were used in order to keep the active site intact. Otherwise, QM and MM water molecules could interchange. The constraints on the water molecules are weak (500 kJ mol^{-1} nm^{-1}). Hence, we do not expect sensible changes if a fully relaxed model is employed, as emphasized in the manuscript.

Table 1S. Gradual (step-by-step) reduction of position restraints and distance constraints while performing QM/MM simulations. The last step (step 7) corresponds to the QM/MM production run. The procedure required a total time of seven weeks on a single processor for each trajectory.

Step	Simulation time [ps]	Position restraint on non-water atoms [kJ mol^{-1} nm^{-1}]	Distance constraint (E123-T127 H-bond)	Position restraint on QM water atoms (only oxygen atoms) [kJ mol^{-1} nm^{-1}]
Step 1	200	5000	Yes	500
Step 2	200	3000	Yes	500
Step 3	200	2000	Yes	500
Step 4	200	1000	Yes	500
Step 5	200	500	Yes	500
Step 6	200	0	No	500
Step 7	1000	0	No	500

ChR2-WT The different DC gate structures of monomer 1 and 2 lead to different structures of the active site. This is not surprising, since a strong coupling between active site and DC gate is expected. The simulation with direct DC gate (monomer 2) shows a stable active site with $RSBH^+$-E123 salt bridge during the entire 2 ns simulation time, which is different from the stable $RSBH^+$-H_2O hydrogen bonding pattern sampled by the simulations with water bridged DC gate (monomer 1). It is unclear whether monomer 1 will also sample salt-bridge structures to a larger extent. Therefore, we decided to discard the monomer 2 structure and extend the sampling of monomer 1. We randomly selected 6 snapshots from the 2 ns trajectory with water bridged DC gate as the starting points for the production QM/MD simulations. Additionally, to get a more accurate description of the hydrogen bond between E123 and T127, we chose a larger QM region

(L-QM), enlarging the S-QM region by including T127. This gave rise to an additional hydrogen link atom for residue T127. New velocities were generated for each simulation.

ChR2-C128T The simulations sample two E123 side chain conformations as shown in Figure 4 of the main text during the 2 ns simulation time, i.e. the E123 side chain either orients towards the cytoplasmic side or moves downwards to the extracellular side. The downshift of E123 breaks the hydrogen bond between E123 and T127 side chains. Note that one MM water molecule stays stable in the active site and forms the water cluster together with the three other QM water molecules. Considering the limitations of MM force fields in describing strong hydrogen bonds in proteins, we chose the same L-QM region on ChR2-C128T as on ChR2-WT. For statistical analysis, we randomly selected 6 snapshots from the 2 ns trajectory of each simulation as the starting points for the production QM/MD simulations.

The results in the main text are based on the simulations with L-QM for both ChR2-WT and ChR2-C128T.

13-*cis*,15-*syn* Retinal The chemical structures of all-*trans* retinal and 13-*cis*, 15-*syn* retinal are shown in Figure 3S (a) and (b), respectively. The *cis* retinal from BR (PDB code: 1X0S) was aligned to the all-*trans* retinal to build the *cis* retinal bound ChR2-WT and ChR2-C128T structures. The $RSBH^+$ has similar positions in the all-*trans* retinal bound complex and the 13-*cis*, 15-*syn* retinal bound complex as shown in Figure 3S (c). The snapshots used for aligning were randomly selected from the all-*trans* retinal trajectories with L-QM. After replacing with *cis* retinal, the protein and the water environment were equilibrated with position restraints (force constant: 1000 kJ mol^{-1} nm^{-1}) on the retinal, K93, E123, T127, D253, and four water molecules involved in the active site hydrogen bonding patterns, to remove the inappropriate atomic conflicts. The equilibration was performed at the MM level of theory using the steepest descent method. The obtained structures were used as starting points for the production QM/MD simulations with the L-QM region.

BR For comparison and to compute the excitation energy shifts, we performed a 1 ns QM/MM simulation of the BR ground state, using a starting structure from our previous study [9]. BR shows a very stable and distinct active site structure, the famous pentagonal water cluster as shown in Figure 1. As we have shown in our earlier publications [58], QM/MM MD simulations only show vibrations around this stable ground states structure, no other local minima occur. Therefore, the amount of sampling to cover this phase-space is much reduced with respect to ChR, where we have shown the large structural heterogeneity. Therefore, the sufficient sampling must assure that the trajectories visit the different local minima sufficiently, in order to converge the statistics. So the BR active site structure is suitably sampled within 1 ns simulation (1000 snapshots).

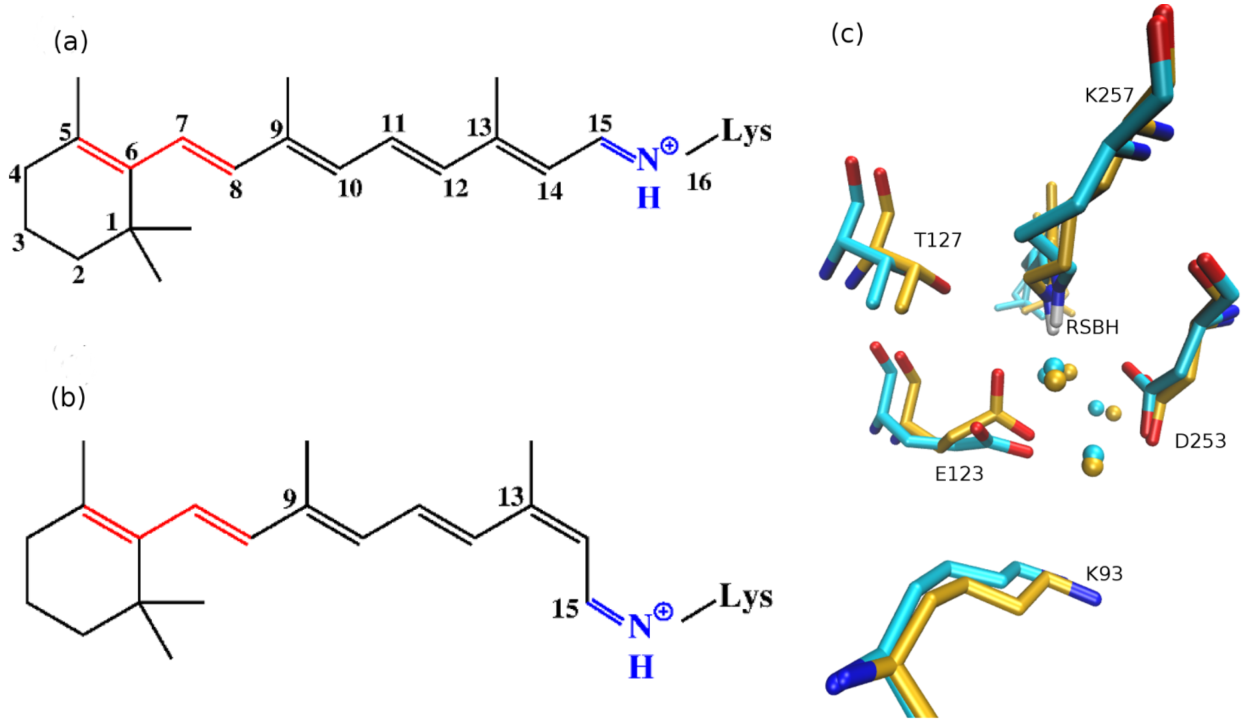

Figure 3S. The chemical structures of (a) all-*trans* retinal and (b) 13-*cis*, 15-*syn* retinal. (c) The alignment of the 13-*cis*, 15-*syn* retinal (orange backbone) to the all-*trans* retinal (cyan backbone) shows that the $RSBH^+$ has similar positions in the protein. The water molecules are shown in orange and cyan balls, respectively.

<u>Simulations at 100K</u> In solid state NMR studies at 100 K [16], the authors observed a shorter distance between $RSBH^+$ and counterion in ChR2 than in BR, which is in conflict with our results at 300 K: BR (3.83 Å) and ChR2 (3.82 Å) have almost the same $RSBH^+$-counterion distance. Nevertheless, a previous study by our group on BR showed a different active site at low temperature with respect to that at room temperature.[38] To get an insight into the temperature dependence of our system, we therefore performed also simulations at 100 K.

We selected 14 snapshots carrying the three hydrogen bonding patterns and two E123 side chain conformations (see main text for details), from the all-*trans* retinal bound ChR2-WT production trajectories. They were used as starting points of 14 independent cooling simulations of 1 ns each. The system was cooled down from 300 K to 100 K within 10 ps, and maintained at 100 K during the remaining simulation time. The same QM/MM setups as for the 300 K production simulations in the main text were adopted.

Table 2S. Summary of structural motifs at 300K (beginning of the simulation) and after the cooling process at 100K.

Trajectory no.	Structural pattern at 300K (beginning of simulation)	Structural pattern at a simulation temperature of 100K
1	E123 up*	E123 up
2	E123 up	E123 up
3	E123 up	E123 up
4	E123 up	E123 up
5	H2O up**	E123 up
6	H2O up	E123 up
7	D253 up***	E123 up
8	D253 up	E123 up
9	D253 up	E123 up
10	H2O up	H2O up<br>E123 up
11	D253 down+	D253 down
12	D253 down	D253 down
13	H2O down++	H2O down<br>D253 down
14	H2O down	H2O down

*E123 up: $-RSBH^{+}\cdots{}^{-}O$-(**E123**) and E123-upward

H2O up: $-RSBH^{+}\cdots$ OH_2;** E123-upward

***D253 up:$-RSBH^{+}\cdots{}^{-}O$-(**D253**); E123-upward

+D253 down: $-RSBH^{+}\cdots{}^{-}O$-(**D253**); E123-downward

++H2O down: $-RSBH^{+}\cdots{}^{-}O$-(**H2O**); E123-downward

Table 3S. The average distance between the $RSBH^+$ nitrogen atom and the center of mass of E123 side chain carboxyl oxygens for the nine trajectories sampling the $-RSBH^+\cdots^-O$-(E123) pattern (unit: Å). For comparison, the same distance averaged on the BR trajectory is 3.83 Å and on ChR2-WT (all-*trans* at 300K) is 3.82 Å.

Trajectories	1	2	3	4	5	6	7	8	9
$RSBH^+$-^-OOC(E123) distance	2.76	2.73	2.79	3.24	3.33	2.99	2.83	3.07	2.75

4. SORCI *vs* OM2/MRCI: Excitation Energies

The SORCI calculations at 50 QM/MM optimized ChR2-WT geometries reveal a major difference in the electronic excited-state description with respect to BR: for both BR and ChR2 (-WT and -C128T) the optically-bright electronic transition is a $^1(\pi,\pi^*)$ transition, but while in BR it is always a $S_0 \rightarrow S_1$ vertical excitation, in ChR2 it corresponds to a $S_0 \rightarrow S_1$ or to a $S_0 \rightarrow S_2$ vertical excitation. In ChR2, S_1 is described − for some of the sampled structures − by a double $^1(\pi,\pi^*)$ transition (*i.e.* a transition involving the promotion of two electrons from bonding to antibonding π orbitals) associated with a low oscillator strength (optically dark electronic transition). The corresponding double $^1(\pi,\pi^*)$ transition corresponds always to S_2 in BR. This inversion between the S_1 and S_2 electronic states may be due to the different active site structure, as already been suggested to explain the hypsochromic absorption shift of ChR2 with respect to BR (as discussed in the main text). Moreover, it may lead to a different description of the *trans*-to-*cis* photoisomerization process. In the main text, the optically bright electronic transition was always taken into account to calculate the absorption spectra. Therefore, all SORCI calculations were performed with three roots (S_0, S_1 and S_2). In order to test the validity of this approach, we show here (Table 2S) the effect of the number of roots on the energy and oscillator strength of the lowest-lying optically bright electronic transition. Two ChR2-C128T models with different active site structures were tested. As it can be seen, the increase of the number of calculated excitation states does not have much influence on the single excitation energy, hence validating our approach.

Table 4S. Single-point excitation energy (E) and oscillator strength (f_{osc}) of the lowest-lying optically bright electronic transition, when including 3 to 6 roots (including the ground state). Two ChR2-C128T geometries were tested. Level of theory: SORCI, complete active space of 12 electrons in 12 orbitals, split-valence basis set def2-SV(P).

**Geometry 1**			**Geometry 2**		
N_{root}	E / eV (nm)	f_{osc}	N_{root}	E / eV (nm)	f_{osc}
3	2.61 (476)	1.83	3	2.98 (416)	1.98
4	2.64 (469)	1.84	4	3.01 (412)	2.05
5	2.69 (461)	1.86	5	3.00 (413)	2.03
6	2.59 (478)	1.79	6	3.00 (412)	1.89

For the sake of comparison, OM2/MRCI calculations were performed at the same 50 QM/MM optimized ChR2-WT geometries as in the SORCI case using the same QM region. The OM2/MRCI calculations employed closed-shell molecular orbitals (MOs) and an active space of 20 electrons in 20 MOs (i.e. the 10 highest occupied MOs and the 10 lowest unoccupied MOs). The reference configurations comprised the closed-shell ground-state configuration and four configurations generated by excitations from the two highest occupied MOs (π, π-1) to the two lowest unoccupied MOs (π*, π*+1), i.e. the single excitations π→π* and π-1→π*+1 as well as the double excitations (π, π)→(π*, π*) and (π, π-1)→(π*, π*+1). All single and double excitations from the reference configurations were included in the MRCI calculations. The same MRCI treatment was applied in the single-point calculations at the snapshot geometries extracted from the QM/MM MD simulations (see main paper).

The cross-correlation function between the SORCI and OM2/MRCI results is shown in Figure 4S, and the simulated absorption spectra are compared in Figure 5S. The relative intensity of each histogram in Figure 5S is calculated as the normalized count of geometries in a defined energy window, multiplied by the average oscillator strength within the same energy window. The energy of each single-point excitation energy can be found in Table 3S. We conclude that a shift of the OM2/MRCI spectra by ca. 0.3 eV can almost quantitatively reproduce the SORCI spectra.

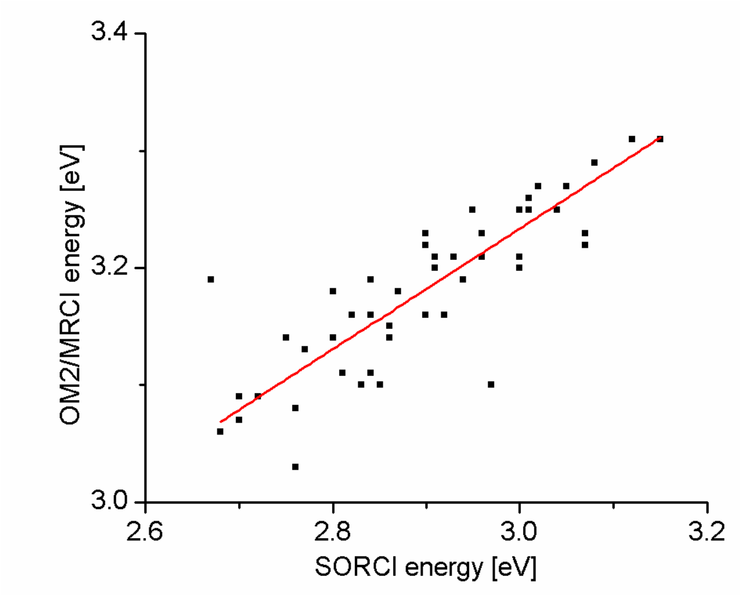

Figure 4S. Cross-correlation function between SORCI and OM2/MRCI excitation energies for the optically-bright electronic transitions. $R^2 = 0.832$.

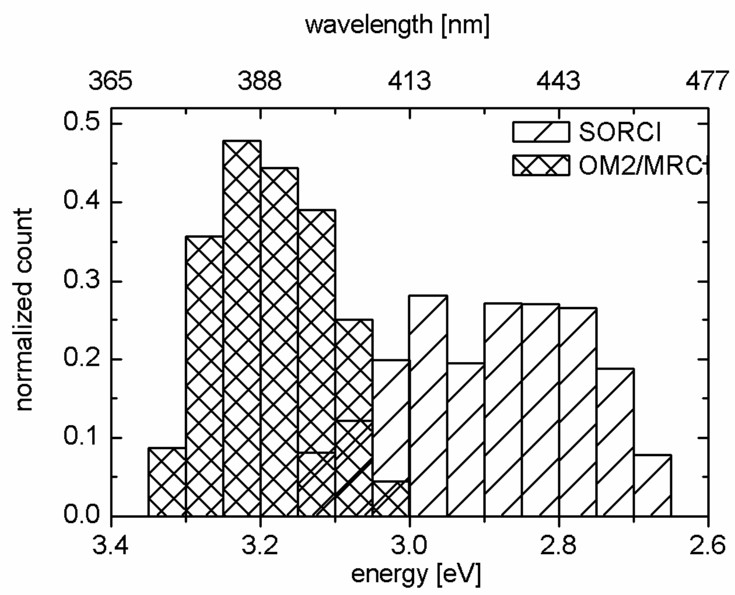

Figure 5S. Simulated ChR2-WT absorption spectra at the SORCI and OM2/MRCI levels of theory. The two histograms are centered at 2.9 and 3.2 eV, respectively (energy shift of ca. 0.3 eV).

Table 5S. Single-point excitation energies from SORCI and OM2/MRCI calculations (unit: eV).

SORCI	OM2/MRCI	ΔE $_{(SORCI-OM2)}$
3,07	3,22	-0,15
2,7	3,07	-0,37
3	3,21	-0,21
3,02	3,27	-0,25
3,01	3,25	-0,24
2,9	3,23	-0,33
3	3,25	-0,25
2,95	3,25	-0,3
2,72	3,09	-0,37
3,04	3,25	-0,21
2,81	3,11	-0,3
2,9	3,16	-0,26
2,67	3,19	-0,52
2,94	3,19	-0,25
2,76	3,08	-0,32
2,84	3,16	-0,32
2,76	3,03	-0,27
2,97	3,1	-0,13
2,85	3,1	-0,25
2,8	3,18	-0,38
2,96	3,21	-0,25
2,77	3,13	-0,36
2,9	3,22	-0,32
2,82	3,16	-0,34
2,96	3,23	-0,27
2,8	3,14	-0,34
2,93	3,21	-0,28
3,08	3,29	-0,21
2,76	3,08	-0,32
2,84	3,11	-0,27
3,12	3,31	-0,19
3,15	3,31	-0,16
2,83	3,1	-0,27
2,86	3,15	-0,29
3,05	3,27	-0,22
2,87	3,18	-0,31
2,91	3,2	-0,29
2,75	3,14	-0,39
2,86	3,14	-0,28
3,07	3,23	-0,16
2,92	3,16	-0,24
3	3,2	-0,2
2,84	3,19	-0,35
2,91	3,21	-0,3
2,68	3,06	-0,38
3,01	3,26	-0,25
2,7	3,09	-0,39

The computed excitation energies depend on the bond length alternation (BLA) of formal carbon-carbon single and double bonds within the polyene chain of the retinal chromophore, both for SORCI and OM2/MRCI (Figure 6S). As expected, the larger the BLA the higher the excitation energy.

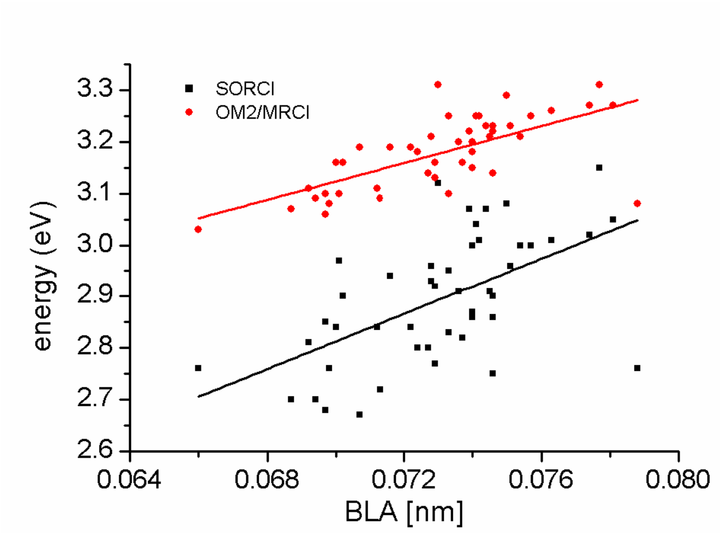

Figure 6S. Dependence of the excitation energy on the retinal bond length alternation (BLA).

Different QM regions were tested with the OM2/MRCI method, in order to establish the most convenient model to be used for the calculation of the OM2/MRCI absorption spectra (Figure 7S). As shown, realistic QM/MM results can be obtained already when only the retinal chromophore is included in the QM region; this was therefore selected as OM2/MRCI QM/MM setup.

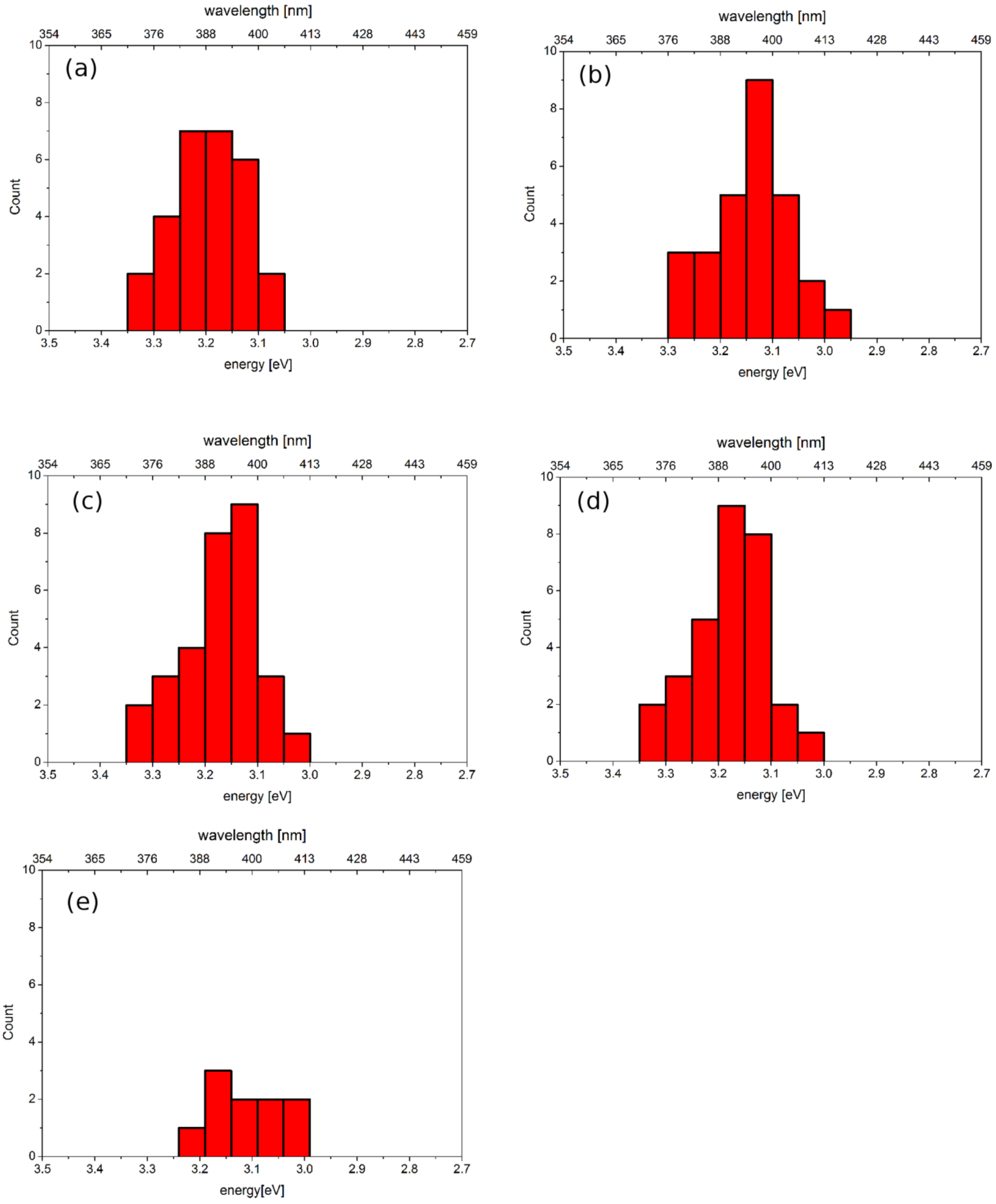

Figure 7S. OM2/MRCI absorption spectra calculated with different QM regions: (a) retinal, four water molecules, E123 and D253 side chains (*i.e.* same as SORCI); (b) only retinal; (c) retinal and D253 side chain; (d) retinal and E123 side chain; (e) retinal and water.

5. Cartesian Coordinates in Ångström of Representative BR and ChR2 Active Site Structures (L-QM region)

BR			
C	26.594540	29.322670	35.907770
H	27.063950	30.238870	35.559420
H	26.476490	28.680350	35.038670
C	27.571830	28.596350	36.912000
O	27.064690	28.037170	37.906610
O	28.799540	28.655520	36.563400
H	25.612640	29.521840	36.329720
C	33.854970	30.951180	38.079040
H	33.278240	30.108470	38.464560
H	34.615920	30.551320	37.413490
C	32.833830	31.795830	37.284960
O	32.383650	32.892950	37.737940
O	32.359450	31.220670	36.215380
H	34.330790	31.522510	38.871780
C	32.714810	28.653280	43.572300
H	32.258810	27.738630	43.954260
H	33.497230	28.367960	42.870390
H	33.160930	29.233610	44.377270
C	31.658430	29.473000	42.846540
H	32.103370	30.390250	42.451690
H	30.888670	29.790000	43.560780
C	30.997700	28.710600	41.700480
H	30.606480	27.751580	42.051310
H	31.724980	28.493030	40.916350
C	29.829700	29.522720	41.119920
H	29.137090	29.780130	41.927750
H	29.285070	28.940540	40.368170
N	30.266980	30.751380	40.479510
H	30.581800	30.639220	39.492940
C	30.213790	31.942110	41.047460
H	29.884030	31.968170	42.090720
C	30.414370	33.139610	40.315670
H	30.590890	33.030980	39.243800
C	30.210440	34.433160	40.802460
C	30.241240	34.760460	42.259100
H	29.469080	35.472300	42.540470
H	31.198030	35.230280	42.494960
H	30.152920	33.876960	42.883780
C	29.961010	35.452630	39.832890
H	29.999130	35.129420	38.792310
C	29.600160	36.768710	40.058580
H	29.574710	37.173020	41.070780
C	29.237890	37.585980	38.958590
H	29.225490	37.080230	37.991490
C	28.914650	38.941380	38.944520
C	28.991020	39.796840	40.172980
H	28.241820	40.584620	40.162100
H	29.968140	40.282590	40.231840
H	28.862690	39.210510	41.079710
C	28.573050	39.490740	37.662790
H	28.616460	38.766180	36.854570
C	28.259650	40.800130	37.370720
H	28.255380	41.499030	38.207750
C	28.008830	41.372880	36.064880
C	27.941290	42.752450	35.954550
C	27.924140	43.669730	37.148800
H	28.770180	43.518040	37.818490
H	27.936900	44.709810	36.832900
H	27.017110	43.519730	37.737510
C	27.823200	43.468000	34.637380
H	26.811990	43.887170	34.569520
H	28.482920	44.343460	34.634360
C	28.082450	42.589410	33.423510
H	27.749100	43.095540	32.512230
H	29.155230	42.402940	33.308190
C	27.329060	41.283640	33.592560
H	27.407350	40.671490	32.689440
H	26.266650	41.510390	33.728510
C	27.835760	40.461490	34.809850
C	26.746820	39.383300	35.062790
H	26.541620	38.849350	34.137920
H	25.820130	39.849840	35.392200
H	27.031210	38.643080	35.802810
C	29.183360	39.801100	34.415620
H	29.075390	39.270610	33.471720
H	29.527510	39.081790	35.152840
H	29.962070	40.551050	34.289900
O	30.813030	29.703120	37.960270
H	31.329920	30.107460	37.232550
H	30.086100	29.165810	37.565660
O	31.684310	31.988830	33.543910
H	30.845360	31.518240	33.713240
H	32.147930	31.977490	34.401220
O	29.817300	30.709430	34.987530
H	29.440090	29.899050	35.378290
H	30.483970	31.080730	35.589390

ChR2, RSBH^{+}···E123			
N	55.21000	23.23000	31.10000
H	55.02000	23.38000	32.06000
C	55.23000	24.53000	30.27000
H	54.34000	24.46000	29.67000
C	55.45000	25.90000	30.95000
H	54.96000	26.05000	31.90000
H	56.53000	26.04000	31.35000
C	55.36000	27.27000	30.09000
H	54.41000	27.38000	29.62000
H	55.45000	28.07000	30.86000
C	56.60000	27.38000	29.15000
H	57.50000	27.08000	29.67000
H	56.45000	26.62000	28.34000
C	56.83000	28.87000	28.56000
H	56.05000	29.27000	27.91000
H	56.89000	29.45000	29.48000
N	58.16000	29.01000	27.90000
H	58.17000	29.91000	27.40000
H	59.00000	28.92000	28.52000
H	58.22000	28.25000	27.20000
C	56.24000	24.16000	28.97000
O	55.89000	24.21000	27.79000
N	50.10000	29.57000	31.19000
H	50.32000	29.69000	30.22000
C	51.23000	29.45000	32.22000
H	50.85000	28.66000	32.85000
C	52.70000	29.43000	31.76000
H	53.00000	28.49000	31.17000
H	52.95000	30.23000	31.07000
C	53.93000	29.48000	32.91000
H	53.75000	28.97000	33.81000
H	54.73000	28.86000	32.46000
C	54.40000	30.87000	33.09000
O	55.19000	31.44000	32.15000

O	54.09000	31.58000	34.11000
C	50.85000	30.53000	33.29000
O	50.72000	30.17000	34.44000
N	49.92000	29.94000	37.29000
H	50.04000	30.28000	36.36000
C	51.05000	30.39000	38.24000
H	50.99000	29.55000	38.92000
C	52.47000	30.56000	37.67000
H	53.23000	30.92000	38.44000
O	52.63000	31.52000	36.51000
H	52.91000	31.01000	35.66000
C	53.22000	29.37000	36.96000
H	54.15000	29.66000	36.40000
H	53.51000	28.68000	37.83000
H	52.52000	28.86000	36.29000
C	50.50000	31.50000	39.14000
O	50.74000	31.43000	40.32000
N	60.16000	35.79000	33.98000
H	60.53000	36.04000	33.08000
C	59.36000	34.46000	34.17000
H	58.54000	34.91000	34.70000
C	58.97000	33.65000	32.92000
H	59.78000	33.18000	32.36000
H	58.32000	32.74000	33.03000
C	58.16000	34.38000	31.65000
O	57.60000	35.47000	31.77000
O	58.23000	33.58000	30.56000
C	60.21000	33.81000	35.31000
O	59.56000	33.26000	36.26000
N	58.62000	33.88000	39.45000
H	59.07000	34.00000	38.57000
C	58.34000	32.46000	39.99000
H	57.81000	32.53000	40.94000
C	57.69000	31.41000	39.07000
H	58.06000	31.45000	38.00000
H	57.94000	30.32000	39.27000
C	56.14000	31.27000	38.73000
H	56.07000	30.37000	38.11000
H	55.62000	30.98000	39.62000
C	55.46000	32.55000	38.14000
H	54.40000	32.55000	38.16000
H	55.77000	33.41000	38.74000
C	55.96000	32.76000	36.67000
H	56.95000	33.31000	36.46000
H	56.12000	31.83000	36.24000
N	54.96000	33.48000	35.84000
H	54.60000	33.12000	34.94000
C	54.50000	34.59000	36.31000
H	54.91000	34.98000	37.24000
C	53.66000	35.43000	35.55000
H	53.21000	35.06000	34.62000
C	53.14000	36.63000	36.05000
C	53.54000	37.10000	37.35000
H	52.68000	37.11000	37.94000
H	54.08000	36.43000	37.91000
H	53.94000	38.04000	37.44000
C	52.33000	37.44000	35.21000
H	52.16000	37.07000	34.19000
C	51.90000	38.70000	35.54000
H	52.30000	39.11000	36.42000
C	51.23000	39.45000	34.52000
H	50.92000	38.83000	33.63000
C	50.78000	40.71000	34.64000
C	51.01000	41.53000	35.91000
H	50.15000	41.78000	36.54000
H	51.69000	41.00000	36.63000
H	51.43000	42.47000	35.62000
C	50.21000	41.43000	33.53000
H	50.34000	40.87000	32.61000
C	49.45000	42.54000	33.54000
H	49.18000	43.03000	34.49000
C	48.82000	43.28000	32.40000
C	48.08000	44.39000	32.71000
C	47.85000	44.92000	34.16000
H	48.80000	45.01000	34.70000
H	47.39000	45.87000	33.88000
H	47.21000	44.20000	34.65000
C	47.28000	45.21000	31.71000
H	46.27000	45.40000	32.05000
H	47.72000	46.26000	31.65000
C	47.36000	44.69000	30.26000
H	46.57000	43.98000	30.10000
H	47.29000	45.42000	29.47000
C	48.74000	44.03000	30.00000
H	48.65000	43.79000	28.90000
H	49.52000	44.78000	30.11000
C	49.05000	42.86000	30.99000
C	50.64000	42.65000	30.74000
H	50.83000	42.89000	29.70000
H	51.18000	43.25000	31.41000
H	50.82000	41.64000	30.83000
C	48.05000	41.67000	30.75000
H	48.32000	40.77000	31.24000
H	47.11000	41.96000	31.07000
H	47.93000	41.32000	29.72000
C	59.73000	32.12000	40.75000
O	59.82000	32.36000	41.97000
O	55.40000	34.26000	33.20000
H	56.33000	34.51000	33.18000
H	55.26000	33.31000	33.09000
O	55.73000	35.46000	29.87000
H	56.52000	35.63000	30.43000
H	55.51000	34.55000	30.04000
O	56.81000	30.72000	34.41000
H	55.87000	30.77000	34.69000
H	56.87000	31.10000	33.51000
O	57.60000	30.94000	31.74000
H	58.09000	31.63000	31.24000
H	56.62000	31.15000	31.94000

ChR2, RSBH^{+}···D253			
N	26.00000	42.79000	31.12000
H	25.71000	42.34000	31.96000
C	26.46000	41.95000	30.00000
H	27.34000	42.48000	29.69000
C	26.85000	40.47000	30.17000
H	27.56000	40.29000	31.04000
H	26.06000	39.78000	30.62000
C	27.38000	39.56000	29.01000
H	28.14000	40.05000	28.42000
H	27.99000	38.74000	29.50000
C	26.37000	39.07000	27.91000
H	26.08000	39.94000	27.33000
H	26.83000	38.27000	27.28000
C	25.14000	38.42000	28.47000
H	25.42000	37.87000	29.35000
H	24.37000	39.03000	28.97000
N	24.47000	37.55000	27.49000
H	23.48000	37.36000	27.74000
H	24.40000	37.99000	26.55000
H	24.92000	36.63000	27.39000
C	25.46000	42.29000	28.76000

O	25.90000	42.46000	27.67000
N	31.62000	36.68000	32.10000
H	31.56000	36.42000	31.14000
C	30.28000	36.80000	32.86000
H	30.53000	37.66000	33.47000
C	28.93000	36.74000	32.13000
H	28.73000	37.44000	31.31000
H	28.78000	35.68000	31.55000
C	27.50000	36.90000	32.88000
H	27.36000	36.14000	33.65000
H	27.51000	37.88000	33.41000
C	26.20000	36.85000	31.91000
O	25.15000	37.36000	32.42000
O	26.22000	36.16000	30.85000
C	30.47000	35.63000	34.07000
O	30.32000	35.90000	35.24000
N	31.14000	36.60000	37.96000
H	31.28000	36.24000	37.04000
C	29.93000	36.18000	38.83000
H	29.82000	37.09000	39.39000
C	28.60000	35.85000	38.13000
H	27.82000	35.48000	38.85000
O	28.63000	34.78000	37.03000
H	29.03000	35.17000	36.25000
C	27.81000	37.06000	37.39000
H	28.34000	37.72000	36.70000
H	27.04000	36.53000	36.82000
H	27.38000	37.76000	38.12000
C	30.49000	35.04000	39.84000
O	30.25000	35.09000	41.06000
N	21.50000	30.41000	33.50000
H	21.55000	30.30000	32.51000
C	22.09000	31.74000	33.94000
H	22.83000	31.33000	34.61000
C	22.60000	32.77000	32.92000
H	21.75000	33.01000	32.15000
H	22.76000	33.82000	33.36000
C	23.89000	32.50000	32.03000
O	24.91000	31.96000	32.54000
O	23.83000	32.89000	30.82000
C	21.00000	32.23000	35.03000
O	21.31000	32.47000	36.20000
N	22.25000	32.40000	38.72000
H	21.75000	32.35000	37.86000
C	22.56000	33.86000	39.12000
H	23.25000	33.65000	39.92000
C	23.16000	34.81000	38.06000
H	22.48000	34.94000	37.15000
H	22.97000	35.90000	38.39000
C	24.73000	34.87000	37.72000
H	24.83000	35.67000	36.98000
H	25.24000	35.20000	38.63000
C	25.39000	33.63000	37.16000
H	26.46000	33.77000	37.30000
H	25.01000	32.78000	37.77000
C	25.04000	33.32000	35.71000
H	23.97000	33.19000	35.49000
H	25.16000	34.27000	35.04000
N	25.87000	32.22000	35.05000
H	25.66000	32.10000	34.08000
C	26.69000	31.39000	35.60000
H	26.79000	31.59000	36.69000
C	27.39000	30.40000	34.86000
H	27.44000	30.63000	33.82000
C	28.08000	29.36000	35.49000
C	27.81000	28.96000	36.93000
H	28.71000	28.58000	37.44000
H	27.54000	29.79000	37.46000
H	26.99000	28.25000	36.92000
C	29.06000	28.57000	34.81000
H	29.33000	28.99000	33.81000
C	29.75000	27.44000	35.16000
H	29.35000	26.95000	36.10000
C	30.66000	26.79000	34.31000
H	30.97000	27.43000	33.45000
C	31.26000	25.59000	34.49000
C	30.96000	24.82000	35.70000
H	30.17000	25.27000	36.37000
H	30.71000	23.83000	35.43000
H	31.85000	24.68000	36.29000
C	32.26000	25.01000	33.58000
H	32.36000	25.62000	32.68000
C	32.78000	23.70000	33.60000
H	32.52000	23.08000	34.50000
C	33.83000	23.21000	32.72000
C	34.58000	22.10000	33.01000
C	34.35000	21.30000	34.35000
H	34.60000	20.23000	34.11000
H	35.09000	21.56000	35.07000
H	33.45000	21.49000	34.90000
C	35.63000	21.53000	32.17000
H	36.36000	21.18000	32.91000
H	35.42000	20.56000	31.63000
C	36.22000	22.49000	31.18000
H	36.86000	23.23000	31.67000
H	36.85000	22.00000	30.54000
C	34.99000	23.19000	30.48000
H	35.32000	23.73000	29.60000
H	34.31000	22.46000	29.99000
C	34.10000	23.99000	31.41000
C	32.80000	24.28000	30.58000
H	32.28000	23.37000	30.37000
H	32.06000	24.83000	31.18000
H	33.06000	24.91000	29.68000
C	34.86000	25.29000	31.70000
H	35.90000	25.10000	32.00000
H	34.92000	25.91000	30.74000
H	34.40000	25.95000	32.44000
C	21.29000	34.36000	39.97000
O	21.27000	34.54000	41.21000
O	26.40000	30.70000	30.62000
H	25.74000	31.06000	31.25000
H	26.19000	31.16000	29.77000
O	27.28000	33.56000	31.09000
H	27.16000	34.49000	30.84000
H	26.68000	33.21000	31.77000
O	25.77000	32.69000	29.08000
H	24.95000	32.64000	29.60000
H	26.52000	33.00000	29.61000
O	25.71000	32.53000	26.53000
H	25.48000	33.33000	26.00000
H	25.68000	32.61000	27.50000

ChR2, RSBH+ $^{+}\cdots OH_2$

N	55.50000	22.66000	31.17000
H	55.62000	22.89000	32.13000
C	55.40000	23.83000	30.16000
H	54.54000	23.37000	29.70000
C	55.13000	25.24000	30.71000
H	54.30000	25.37000	31.36000
H	55.91000	25.55000	31.49000
C	55.11000	26.58000	29.88000
H	54.52000	26.59000	28.96000

H	54.70000	27.34000	30.61000
C	56.52000	27.13000	29.43000
H	57.17000	27.35000	30.28000
H	57.00000	26.32000	28.74000
C	56.47000	28.35000	28.61000
H	55.84000	28.24000	27.64000
H	55.94000	29.13000	29.09000
N	57.79000	28.88000	28.13000
H	57.52000	29.78000	27.75000
H	58.48000	28.97000	28.89000
H	58.18000	28.28000	27.37000
C	56.50000	23.50000	28.99000
O	56.19000	23.66000	27.79000
N	49.76000	29.68000	31.24000
H	50.00000	29.98000	30.32000
C	50.97000	29.34000	32.14000
H	50.52000	28.51000	32.65000
C	52.38000	29.10000	31.58000
H	52.47000	28.17000	30.99000
H	52.84000	29.88000	30.98000
C	53.60000	28.84000	32.71000
H	53.17000	28.31000	33.62000
H	54.29000	28.14000	32.22000
C	54.33000	30.09000	33.11000
O	55.38000	30.37000	32.37000
O	54.00000	30.76000	34.11000
C	50.82000	30.49000	33.36000
O	50.76000	30.05000	34.54000
N	49.97000	29.66000	37.29000
H	50.12000	29.80000	36.31000
C	51.13000	30.06000	38.19000
H	51.11000	29.27000	38.93000
C	52.54000	30.27000	37.60000
H	53.41000	30.47000	38.49000
O	52.70000	31.45000	36.60000
H	52.79000	31.06000	35.67000
C	53.29000	29.06000	36.83000
H	54.14000	29.44000	36.22000
H	53.57000	28.11000	37.37000
H	52.58000	28.55000	36.15000
C	50.66000	31.20000	39.22000
O	50.95000	31.24000	40.40000
N	59.92000	35.88000	34.35000
H	60.09000	35.99000	33.37000
C	59.40000	34.42000	34.63000
H	58.50000	34.75000	35.12000
C	59.15000	33.46000	33.46000
H	60.12000	33.06000	33.01000
H	58.57000	32.61000	33.74000
C	58.24000	33.82000	32.19000
O	57.55000	34.87000	32.18000
O	58.12000	32.86000	31.31000
C	60.32000	34.00000	35.93000
O	59.88000	33.88000	37.03000
N	58.59000	33.81000	39.74000
H	59.22000	34.05000	39.00000
C	58.32000	32.30000	40.14000
H	57.67000	32.52000	40.97000
C	57.80000	31.29000	39.11000
H	58.33000	31.26000	38.15000
H	58.01000	30.19000	39.43000
C	56.18000	31.06000	38.71000
H	56.15000	30.19000	38.00000
H	55.65000	30.89000	39.69000
C	55.52000	32.29000	38.10000
H	54.51000	32.38000	38.27000
H	55.93000	33.15000	38.46000
C	55.84000	32.35000	36.59000
H	56.88000	32.22000	36.53000
H	55.49000	31.40000	36.12000
N	55.20000	33.34000	35.78000
H	55.39000	33.23000	34.81000
C	54.53000	34.35000	36.28000
H	54.43000	34.38000	37.35000
C	53.96000	35.34000	35.52000
H	54.11000	35.33000	34.43000
C	53.49000	36.52000	36.10000
C	53.66000	36.90000	37.51000
H	54.43000	37.70000	37.57000
H	52.86000	37.36000	38.06000
H	54.06000	36.01000	38.07000
C	52.79000	37.44000	35.23000
H	52.76000	37.08000	34.18000
C	52.05000	38.49000	35.72000
H	51.87000	38.68000	36.81000
C	51.44000	39.38000	34.81000
H	51.52000	39.01000	33.78000
C	50.91000	40.65000	34.99000
C	50.93000	41.25000	36.33000
H	49.93000	41.35000	36.73000
H	51.44000	40.52000	36.95000
H	51.48000	42.21000	36.46000
C	50.44000	41.32000	33.76000
H	50.58000	40.71000	32.84000
C	49.97000	42.61000	33.75000
H	50.04000	43.29000	34.59000
C	49.34000	43.30000	32.57000
C	48.80000	44.50000	32.82000
C	48.94000	45.33000	34.07000
H	48.81000	44.81000	34.96000
H	49.90000	45.82000	34.05000
H	48.08000	46.07000	34.08000
C	47.87000	45.21000	31.84000
H	47.00000	45.52000	32.42000
H	48.37000	46.07000	31.51000
C	47.41000	44.36000	30.64000
H	46.59000	43.71000	31.03000
H	47.22000	45.09000	29.81000
C	48.56000	43.51000	30.18000
H	48.25000	42.93000	29.23000
H	49.34000	44.07000	29.80000
C	49.11000	42.59000	31.27000
C	50.51000	42.08000	30.69000
H	51.14000	42.92000	30.49000
H	51.09000	41.46000	31.34000
H	50.41000	41.71000	29.71000
C	48.18000	41.35000	31.45000
H	47.17000	41.74000	31.48000
H	48.32000	40.77000	30.61000
H	48.34000	40.68000	32.31000
C	59.66000	31.99000	40.99000
O	59.78000	32.07000	42.20000
O	55.63000	32.96000	33.10000
H	56.17000	33.58000	32.57000
H	55.43000	32.01000	32.73000
O	56.13000	34.85000	29.96000
H	55.76000	33.98000	30.22000
H	56.87000	35.12000	30.64000
O	57.41000	30.60000	34.52000
H	57.74000	30.12000	33.78000
H	56.50000	30.84000	34.17000
O	58.16000	30.22000	31.77000

H	58.21000	31.21000	31.77000
H	57.17000	30.17000	31.77000

6. Metadynamics

The dihedral angle around the C_β-C_γ bond was used as the collective variable (i.e. the reaction coordinate). The parameters were set as follows: Gaussian height of 0.2 kcal/mol, Gaussian width of 0.2 rad, Gaussian depositing frequency of 2 ps (1000 time steps), bias factor of 4, and temperature of 300 K. The convergence of the metadynamics simulation was evaluated by comparing the free energy as a function of the collective variable at different times, as shown in Figure 8S. The similar profiles obtained after 85 ns indicates a good convergence of the simulation.

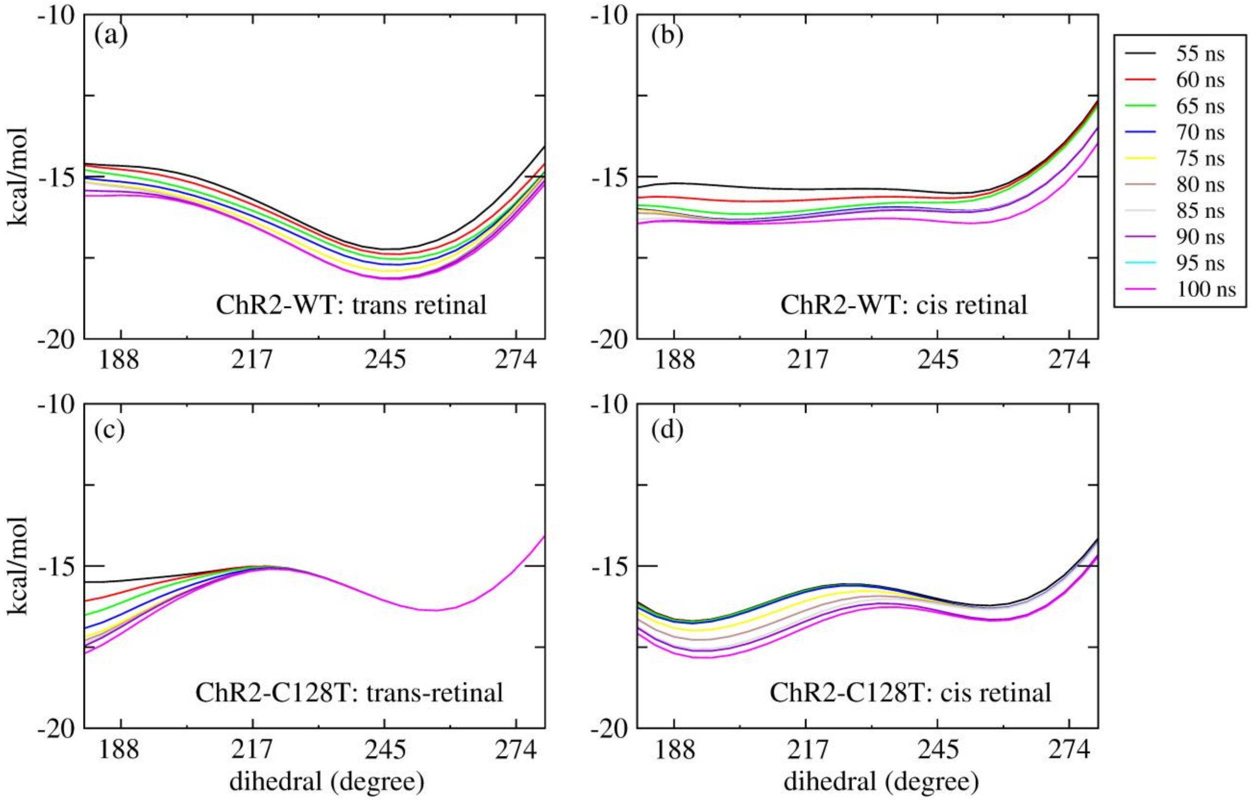

Figure 8S. The free energy as a function of the dihedral angle around the C_β-C_γ bond of E123 every 5 ns along the 100 ns well-tempered metadynamics simulation. (a) ChR2-WT bound with all-*trans* retinal; (b) ChR2-WT bound with 13-*cis*, 15-*syn* retinal; (c) ChR2-C128T bound with all-*trans* retinal; (d) ChR2-C128T bound with 13-*cis*, 15-*syn* retinal.

7. Gaussian Fit Parameters

The analytic fitted Gaussian function parameters used to convolute the OM2/MRCI histograms, shown in Figures 7–9 of the main text, are listed in Tables 4S.

Table 6S. Fitting parameters used to convolute the OM2/MRCI histograms into Gaussian functions of Figures 7–9 in the main text.

	Fit function: y = -A0*exp(A2*(A1-x)^2)			
	A0	A1	A2	Fitting coefficient: R^2
BR	-0.246117	2.62478	-24.7658	0.997614
ChR2-WT: *trans*+*cis* retinals, total	-0.178744	3.19532	-14.8734	0.998295
ChR2-WT: *trans*+*cis* retinals, RSBH-E123	-0.0781826	3.24628	-18.9074	0.996242
ChR2-WT: *trans*+*cis* retinals, RSBH-D253	-0.0528687	3.14634	-13.1284	0.996214
ChR2-WT: *trans*+*cis* retinals, RSBH-H_2O	-0.154118	3.16907	-15.3466	0.995477
ChR2-WT: *trans*+*cis* retinals, E123-upward	-0.125309	3.24356	-18.7595	0.998637
ChR2-WT: *trans*+*cis* retinals, E123-downward	-0.0683909	3.10066	-15.5577	0.996431
ChR2-WT: *trans* retinal, total	-0.19488	3.20365	-16.9763	0.998440
ChR2-WT: *trans* retinal, RSBH-E123	-0.0976391	3.23267	-18.5911	0.997837
ChR2-WT: *trans* retinal, RSBH-D253	-0.0142415	3.18572	-19.7828	0.972275
ChR2-WT: *trans* retinal, RSBH-H_2O	-0.0864315	3.17354	-16.3061	0.995963
ChR2-WT: *trans* retinal, E123-upward	-0.147067	3.23107	-18.8384	0.997145
ChR2-WT: *trans* retinal, E123-downward	-0.0551499	3.13108	-16.9933	0.994725
ChR2-WT: *cis* retinal, total	-0.163051	3.1837	-12.7511	0.997197
ChR2-WT: *cis* retinal, RSBH-E123	-0.0976391	3.23267	-18.5911	0.997837
ChR2-WT: *cis* retinal, RSBH-D253	-0.0142415	3.18572	-19.7828	0.972275
ChR2-WT: *cis* retinal, RSBH-H_2O	-0.0864315	3.17354	-16.3061	0.995963
ChR2-WT: *cis* retinal, E123-upward	-0.147067	3.23107	-18.8384	0.997145

ChR2-WT: *cis* retinal, E123-downward	-0.0551499	3.13108	-16.9933	0.994725
ChR2-C128T: *trans*+*cis* retinals, total	-0.192937	3.05524	-15.2363	0.998059
ChR2-C128T: *trans*+*cis* retinals, RSBH-E123	-0.00992061	3.19575	-20.4072	0.973085
ChR2-C128T: *trans*+*cis* retinals, RSBH-D253	-0.0457847	3.06655	-16.2952	0.994600
ChR2-C128T: *trans*+*cis* retinals, RSBH-H_2O	-0.140947	3.04233	-15.5087	0.999122
ChR2-C128T: *trans*+*cis* retinals, E123-upward	-0.0175266	3.17342	-18.2792	0.984749
ChR2-C128T: *trans*+*cis* retinals, E123-downward	-0.179718	3.04509	-15.7911	0.997950
ChR2-C128T: *trans* retinal, total	-0.202365	3.08519	-15.7886	0.998103
ChR2-C128T: *trans* retinal, RSBH-E123	-0.0196258	3.19547	-20.1408	0.970591
ChR2-C128T: *trans* retinal, RSBH-D253	-0.038766	3.09045	-17.2507	0.995595
ChR2-C128T: *trans* retinal, RSBH-H_2O	-0.149439	3.06946	-16.2166	0.995911
ChR2-C128T: *trans* retinal, E123-upward	-0.0316859	3.18153	-18.8221	0.984326
ChR2-C128T: *trans* retinal, E123-downward	-0.176413	3.06918	-16.4274	0.998190
ChR2-C128T: *cis* retinal, total	-0.189411	3.02266	-15.711	0.997310
ChR2-C128T: *cis* retinal, RSBH-E123	-0.00044238	3.21333	-128.534	0.836400
ChR2-C128T: *cis* retinal, RSBH-D253	-0.0530368	3.04958	-15.7043	0.985150
ChR2-C128T: *trans* retinal, RSBH-H_2O	-0.13747	3.01213	-16.0913	0.996802
ChR2-C128T: *trans* retinal, E123-upward	-0.0037326	3.09766	-15.5254	0.855565
ChR2-C128T: *trans* retinal, E123-downward	-0.186148	3.02121	-15.8074	0.997509

8. References

[1S] Berendsen, H. J. C.; Postma, J. P. M.; van Gunsteren, W. F.; Di Nola, A.; Haak, J. R. *J. Chem. Phys.* 1984, 81, 3684-3690.
[2S] Parrinello, M.; Rahman, A. *J. Chem. Phys.* 1982, 76, 2662-2666.
[3S] Nosé, S. *J. Chem. Phys.* 1984, 81, 511-519.
[4S] Hoover, W. G. *Phys. Rev. A* 1985, 31, 1695-1697.
[5S] Nack, M.; Radu, I.; Gossing, M.; Bamann, C.; Bamberg, E.; von Mollard, G. F.; Heberle, J. *Photochem. Photobiol. Sci.* 2010, 9, 194-198.
